# Relationship between Age/Gender-Induced Survival Changes and the Magnitude of Inflammatory Activation and Organ Dysfunction in Post-Traumatic Sepsis

**DOI:** 10.1371/journal.pone.0051457

**Published:** 2012-12-12

**Authors:** Susanne Drechsler, Katrin Weixelbaumer, Pierre Raeven, Mohammad Jafarmadar, Anna Khadem, Martijn van Griensven, Soheyl Bahrami, Marcin Filip Osuchowski

**Affiliations:** Ludwig Boltzmann Institute for Experimental and Clinical Traumatology in the Trauma Research Center of Allgemeine Unfallversicherungsanstalt, Vienna, Austria; University of Cincinnati, United States of America

## Abstract

Age/gender may likely influence the course of septic complications after trauma. We aimed to characterize the influence of age/gender on the response of circulating cytokines, cells and organ function in post-traumatic sepsis. We additionally tested whether post-traumatic responses alone can accurately predict outcomes in subsequent post-traumatic sepsis. A mouse 2-hit model of trauma/hemorrhage (TH, 1^st^ hit) and cecal ligation and puncture (CLP, 2^nd^ hit) was employed. 3, 15 and 20 month (m) old female (♀) and male (♂) CD-1 mice underwent sublethal TH followed by CLP 2 days later. Blood was sampled daily until day 6 post-TH and survival was followed for 16 days. To compare general response patterns among groups, we calculated two scores: the inflammatory response (including KC, MIP-1α, TNFα, MCP-1, IFNγ, IL-1β,-5,-6,-10) and the organ dysfunction score (Urea, ALT, AST and LDH). Moreover, mice were retrospectively divided into survivors (SUR) and dying (DIE) based on post-CLP outcome. In general, females survived better than males and their survival did not correspond to any specific estrus cycle phase. *Pre-CLP phase*: the post-TH inflammatory score was weakest in 3 m♂ but there were no changes among remaining groups (similar lack of differences in the organ dysfunction score). TH induced a 40% increase of IFNγ, MIP-1α and IL-5 in 15 m♂ SUR (vs. DIE) but predictive accuracy for post-CLP outcomes was moderate. *Post-CLP phase*: while stable in males, inflammatory response score in 15 m and 20 m females decreased with age at day 1 and 2 post-CLP. SUR vs. DIE differences in inflammatory and organ dysfunction score were evident but their magnitude was comparable across age/gender. Nearly identical activation of the humoral inflammatory and organ function compartments, both across groups and according to sepsis severity, suggests that they are not directly responsible for the age/gender-dependent disparity in TH-CLP survival in the studied young-to-mature population.

## Introduction

Trauma is one of the leading causes of morbidity and mortality in the young and adult populations in the United States and Europe [1], [2]. Traumatic insults are typically accompanied by a blood loss of different magnitude, and frequently followed by a hemorrhagic shock in more severe cases [3]. While trauma induces the release of local and systemic mediators to initiate tissue repair and support hemostasis and wound healing, primary hemodynamic responses aim to compensate the blood loss by an increase of heart rate and vascular resistance to avoid a state of hypovolemic shock [3], [4]. These mechanisms represent a major challenge to the patient’s immuno-inflammatory system and can potentially impair his/her immune functions predisposing such an individual towards secondary complications including sepsis. The incidence of septic complications following major trauma varies between 2% and 30% [4], [5]. In trauma patients, sepsis is the third most frequent cause of morbidity and mortality after multi organ dysfunction syndrome (MODS) and acute lung injury [6]. The correlation between injury severity and increased risk of developing sepsis remains strong [5], and the ICU admission rates and mortality (94% and 40%) are significantly higher in trauma patients who develop sepsis compared to their non-septic counterparts [5].

Mice are a commonly accepted and frequently used animal species for the investigation of the effects of trauma/hemorrhage and/or sepsis on the immune response [7], [8]. Preclinical studies in rodent models have shown that both gender and age can strongly modulate immune functions after trauma and hemorrhage. Kahlke et al. demonstrated that, following traumatic hemorrhagic shock in young male mice, release of IL-1β and IL-6 by splenic and peritoneal macrophages was depressed, while production of IL-10 was enhanced compared to young female mice [9]. The opposite was true in aged animals: an enhanced response of both peritoneal macrophages and T-lymphocytes was demonstrated in aged males compared to aged females [10]. Moreover, young male mice showed a significantly decreased survival compared to proestrus females (6% vs. 67%) when challenged with secondary sepsis 24 h after trauma and hemorrhage [11].

In human patients, however, the prevalence and role of the gender/age in the immuno-inflammatory responses provoked by traumatic/septic events remains controversial. Female gender (independent of age) was associated with lower risk for MODS and nosocomial infections (43% and 23%) after injury and hemorrhagic shock [12], while Schroeder et al. demonstrated a significantly better prognosis for women with surgical sepsis compared to male patients [13]. A study with 143 polytrauma patients demonstrated a reduction of MODS (by 12% and 15%) and sepsis incidence (but not mortality) in females (predominantly premenopausal) compared to age-matched males [14]. In contrast, Rappold et al. (approx. 2250 adults, retrospective study) concluded that female gender does not save blunt trauma patients from secondary complications (including sepsis) and mortality [15], while another report (709 patients, prospective study) demonstrated a 10% increase of mortality in septic women compared to septic men [16].

In both trauma and sepsis, the body’s immuno-inflammatory system frequently produces the systemic inflammatory response syndrome (SIRS) - an excessive systemic (and simultaneous) release of pro- and anti-inflammatory mediators that may be later followed by immunosuppression. These events often lead to the development of MODS with a mortality ranging between 20–75%, depending on the number of failing organs [17], [18]. Yet, despite of those multidirectional inflammatory responses, the search for biomarker(s) with a capacity to accurately predict secondary infections and/or their outcome in heterogeneous trauma/hemorrhage patients has been largely ineffective: C-reactive protein, typically elevated within 48 h of trauma, fails to detect early infections better than other potential markers [19], [20]. Post-traumatic procalcitonin increases show no correlation to later adverse outcomes [21], while TNF-α has failed to predict mortality in trauma patients whatsoever [22], [23]. Only the rise of circulating lactate and IL-6 has been to some extent associated to development of MODS, injury severity and subsequent mortality in trauma [24]–[26]. Recently, NT-proCNP, the N-terminal fragment of the C-type natriuretic peptide precursor, has been found to predict septic complications in trauma patients without traumatic brain injury [27] and to correlate with outcome in septic patients [28]. The above underlines the necessity for systematic elucidation of the general fluctuations in inflammatory and organ function patterns among different age and gender groups – specifically regarding the exact role initial trauma and hemorrhage play in the evolution of subsequent septic responses. Therefore, in the present study we aimed to: 1) simultaneously investigate the impact of age and gender on the inflammatory and organ (dys)functions in different phases of post-traumatic sepsis in CD-1 mice, and 2) assess the predictive capacity of post-traumatic responses for secondary sepsis outcomes in mice of different age and gender.

## Materials and Methods

### Animals

Outbred, Hsd:ICR (CD-1)®, SPF mice (total n = 234), from Harlan Laboratories (Udine, Italy) were used for all experiments. Animals were divided into the following experimental groups: 1) “young” groups including 3 months old females (average weight 30 g, n = 43) and males (40 g, n = 25), 2) “middle-age” groups including 15 months old females (50 g, n = 53) and males (50 g, n = 50), and 3) “mature” groups including 20 months old female (48 g, n = 32) and male (52 g n = 31) mice. All animals were allowed to acclimatize to their new environment for at least one week after their delivery. In order to precisely adhere to the stated age, we kept the inter-group deviations within one week of age.

Female mice were kept in groups of five animals per Type-III cage, while males were kept single. All mice were housed on a 12 h light-dark diurnal cycle with controlled temperature (21–23°C) and provided with standard rodent diet and water *ad libitum* throughout all experiments. Cages were enriched with houses, wood wool for nesting as well as wooden boards, tunnels and small blocks for gnawing (Abedd Lab & Vet Service, Vienna, Austria) to facilitate natural behavior prior to and throughout the experimentation.

### Ethics Statement

All animal procedures were approved by the Viennese (Austria) legislative committee (Animal Use Proposal Permission no: 000794/2009/13) and conducted according to National Institute of Health guidelines.

Due to the severe nature of the TH-CLP model additionally complicated by diverse age/gender groups, a particular focus was put on monitoring of animals in the study to minimize suffering within the frames of the experimental design. All mice enrolled in the study were kept in the institute’s small in-house animal facility to enable optimal monitoring: the overall health status was checked by trained professionals (i.e. DVMs and/or MDs) at least three times per day and more (typically every 2–3 h) whenever a mouse’s condition deteriorated (defined by, e.g. decreased activity, progressing hypothermia, rapid weight gain; [29]). To further enhance monitoring capacity and ensure maximal data reproducibility, all experiments were conducted in small animal groups (typically 10–20 per cycle).

Given that the objective of our study was to compare immuno-inflammatory and organ (dys)function status between non-surviving and surviving mice, the time of septic death was the most critical endpoint. This precluded us to follow typical humane endpoints as they are inadequate and too imprecise in the critical care disease models such as sepsis, shock, trauma and burns [29]: sacrificing mice based on typically accepted parameters (e.g. temperature, weight loss) in any of those late-stage disease studies may be premature and falsify the results. For example, in the CLP model, a weight gain not its loss is more predictive of impending death and hypothermia of 30°C has a positive predictive value of only 56% for death within 24 h [29], while we observed cases of full recovery in septic mature male mice displaying body temperature of ≤28°C for up to three post-CLP days (unpublished observations).

Therefore, as suggested by Nemzek et al. [29] we followed a more precise, empirically established, set of guidelines that offered, although a very narrow, yet a feasible (due to the frequent monitoring) window of opportunity for induction of death that did not hinder this study’s experimental setup. Specifically, mice were killed only upon signs of imminent demise (i.e. inability to maintain upright position/ataxia/tremor and prolonged/deep hypothermia and/or agonal breathing) by using deep inhalation anesthesia (isoflurane, Forane®) followed by an overdose of barbiturate (thiopental, Thiopental Sandoz ®).

### Two-hit Model: Trauma/hemorrhage Followed by Sepsis

**Figure 1:**
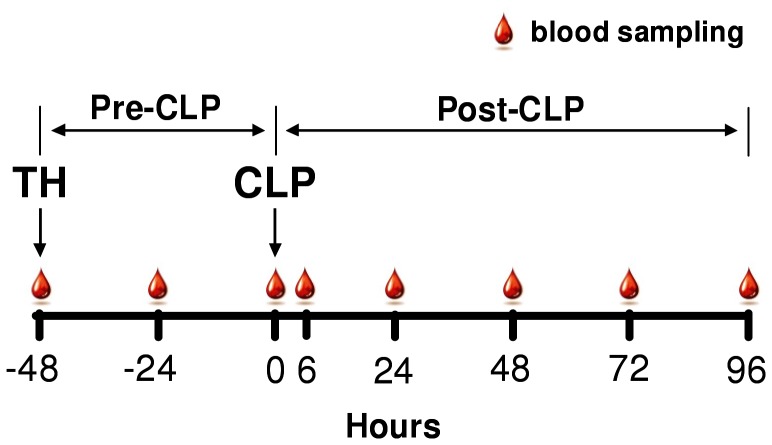
Schematic of the two-hit model combined with the repetitive blood sampling approach. 3, 15 and 20 month old female and male mice were subjected to trauma and hemorrhage (TH) followed by polymicrobial CLP sepsis. The observation time-span was divided into the pre-and post-CLP phases. Starting at TH, a 20 µl blood sample was collected daily (indicated by a single blood drop) until day 7 post-TH (day 5 post-CLP) from each animal (an additional blood sample was collected at 6 h post-CLP).

Figure 1 shows experimental design of the two-hit approach. The first hit was modified based on the previously described protocol [30], and consisted of trauma immediately followed by sublethal hemorrhagic shock (TH). Trauma was defined as an unilateral, non comminuted femoral fracture with local soft tissue damage realized with a custom-built, forcipate device, whereas hemorrhagic shock was induced by drawing 40% of the total blood volume (calculated as 6% of body weight) via retro-orbital puncture. Mice were resuscitated with four times of the shed blood volume. The first ¼ included analgesia (0.05 mg/kg buprenorphine, Buprenovet®) and was administered subcutaneously immediately post-TH, the remaining ¾ after 1 h by another subcutaneous injection of Ringer’s solution.

For the second hit, mice were subjected to cecal ligation and puncture (CLP; 48 h after TH) following the original protocol by Wichterman et al. [31] with modifications specified elsewhere [32], [33]. Based on our previous study, we had chosen the 17-gauge needle size as in our laboratory it induces approximately 50% mortality in 3 month old females on day 5 after TH-CLP (the calibration group) [30]. In brief, after opening the abdominal cavity via midline laparatomy, cecum was exposed, ligated and punctured twice with a 17-gauge needle. Abdomen was closed with two single button sutures and Histoacryl® skin adhesive. To maximally simulate ICU treatment protocol of human patients, mice received subcutaneous wide-range antibiotic therapy (25 mg/kg imipenem, Zienam®) and fluid resuscitation (1 ml Ringer’s solution) with analgesic (0.05 mg/kg buprenorphine, Buprenovet®) twice daily for five consecutive days post-CLP. We chose the subcutaneous over peritoneal route as it is least stressful to animals. Additionally, the effects of peritoneal application of antibiotics in CLP can differ among septic individuals due to a developing inflammatory process followed by abdominal compartmentalization. From day five until the end of the experiment mice were provided with metamizol (6 mg/kg Novalgin®) via drinking water.

All surgical procedures were performed in the morning, between 8–10 am. Mice were anesthetized with isoflurane (induction 3%, maintenance 1.5% Forane®) and survival was monitored for 16 days post-TH. Given the aim of the experiment, sham surgeries were not performed to reduce the total number of mice in the study. All surviving animals were killed with deep-inhalation anesthesia followed by an overdose of barbiturate at the end of experiments.

### Blood Sampling

Starting immediately prior to trauma/hemorrhage (−48 h time-point, Fig. 1 scheme), 20 µl of blood was collected via facial vein (*vena submandibularis*) puncture (with 23G needle) from each animal every 24 h (including an additional sample at 6 h post-CLP) until day 5 post-CLP as previously described by Weixelbaumer et al. [34]. All samples were immediately diluted 1∶10 in PBS rinsed with ethylenediaminetetraacetic acid (EDTA) (diluted 1∶50). After centrifugation (1000×g, 5 min, 22°C), 180 µl of plasma was removed and stored at −80°C for further analysis.

### Complete Blood Count

After removing plasma, the remaining blood pellet was resuspended with 180 µl Cell-Dyn buffer with EDTA and a complete blood count (CBC) with differential was performed with a CellDyn 3700 counter (Abbott Laboratories, Illinois, USA).

### Cytokine Assay

Interleukin (IL)-1β, IL-10, IL-5, IL-6, interferon (IFN)-γ, tumor necrosis factor (TNF)–α, macrophage inflammatory protein (MIP)-1α, chemokine ligand (KC; CXCL-1) and monocyte chemoattractant protein-1 (MCP-1) were analyzed from plasma samples using FlowCytomix™ Multiplex Kits (eBioscience, USA) according to manufacturer protocol.

### Metabolic and Organ Function Parameters

Urea nitrogen (urea), glucose, lactate dehydrogenase (LDH), alanin transaminase (ALT) and aspartate transaminase (AST) were analyzed in plasma samples with a Cobas c111 analyzer (Roche, Switzerland).

### Vaginal Cytology

Mice were restrained with one hand and vagina was flushed 5 times using a pipette containing 10 µl of normal saline. Collected fluid was then placed on a microscope slide and allowed to dry. Next, slides were stained with Diff-Quick (Medion, Switzerland) staining method and analyzed by two independent evaluators.

### Statistical Analysis

The samples size estimation was calculated by power analysis with StatMate (GraphPad, Software Inc., San Diego, USA) prior to the study. We used the young groups as a calibrator for survival experiments and set up the fraction of surviving subjects at 0.5 by day 28 post-CLP. Aiming to detect inter-gender change in survival proportion of approx. 0.3 using the significance level (alpha) = 0.05 (two-tailed) and power = 80%, an estimated sample size of 40 mice per arm was defined. For clarity, n group sizes are displayed in each figure/bar.

16-day survival curves were plotted using Kaplan-Meier method. Data were tested for normality using D’Agostino and Pearson and Shapiro-Wilk tests. For statistical analysis the experiment was divided into the pre- and post-CLP phase (Fig. 1 scheme).

Composite inflammatory response and organ dysfunction scores were generated based on a calculation protocol used in our two recent studies [35]; [36]. Figure S1 provides step-by-step explanation how the scores were calculated. For each age/gender group, all cytokine values (each individually) were normalized to the median value of that specific cytokine. First, the median value was selected from the two age/gender groups with the highest and lowest mean concentrations of this specific cytokine at 24 h post-TH and 24 h post-CLP (the two time points when the recorded changes in cytokine and OF responses were well pronounced). This selected median value was then used for normalization of all other values of this specific cytokine to produce individual score values for all mice in enrolled age/gender groups. Next, all these individual scores were tallied together according to age and gender, generating a score data set for each of those groups. This data set was then averaged producing an average inflammatory score (for this specific cytokine) for each age/gender group. Identical steps were performed for all remaining cytokines resulting in a total of nine average score values (each average score value representing one cytokine we measured) for each age and gender group. These inflammatory scores were then compared among age and gender groups. The same approach was used to calculate average organ dysfunction scores using urea, ALT, AST, and LDH.

Differences within the same age or gender group were evaluated by Student’s t test (normally distributed data) with Welch’s correction if needed or Mann-Whitney test (skewed data). Pre-CLP reference values for organ function were recalculated to match the post-CLP scores. For further analyses of CBC, organ function parameters and cytokine release mice were retrospectively divided into either surviving (SUR, alive by day 14 post-CLP) or dying (DIE, died between day 1–14 post-CLP). SUR vs. DIE differences within one age/gender group (each time-point separately) were assessed by either Student’s t test (with Welch’s correction if needed) or Mann-Whitney test. Comparisons between SUR and DIE mice were restricted to sampling time points up to 48 h post-CLP due to the loss of animals in the DIE groups. To determine the outcome prognostic accuracy of single cytokines measured pre-CLP, we used the receiver operating characteristic (ROC) curve defined by the area under the curve (AUC). The accuracy of the ROC-AUC test is scaled as follows: 0.9–1, excellent; 0.8–0.9, good; 0.7–0.8, fair; 0.6–0.7, poor, and <0.6, not useful.

Inflammatory response and organ dysfunction scores recorded during either pre-or post-CLP phase are presented as bars with SD. Individual cytokines, glucose and CBCs are presented as box and whiskers (Min, IQR 2, Median, IQR 3, Max), while post-CLP data were plotted as mean ± SEM. Baseline cytokine levels assessed from pure plasma below the detection limit were given a value equal to half of the lower limit of detection (LLD), while all other 1∶10 diluted samples were assigned a value equal to 10 times half of the LLD. The LLD ranged between 5–69 pg/ml. Statistical tests were carried out using Prism 5 (GraphPad Software Inc., San Diego, USA) and the level of significance was defined at p<0.05.

## Results

### Pre-CLP phase

#### Age and gender did not modify the global inflammatory response induced by trauma/hemorrhage

**Figure 2:**
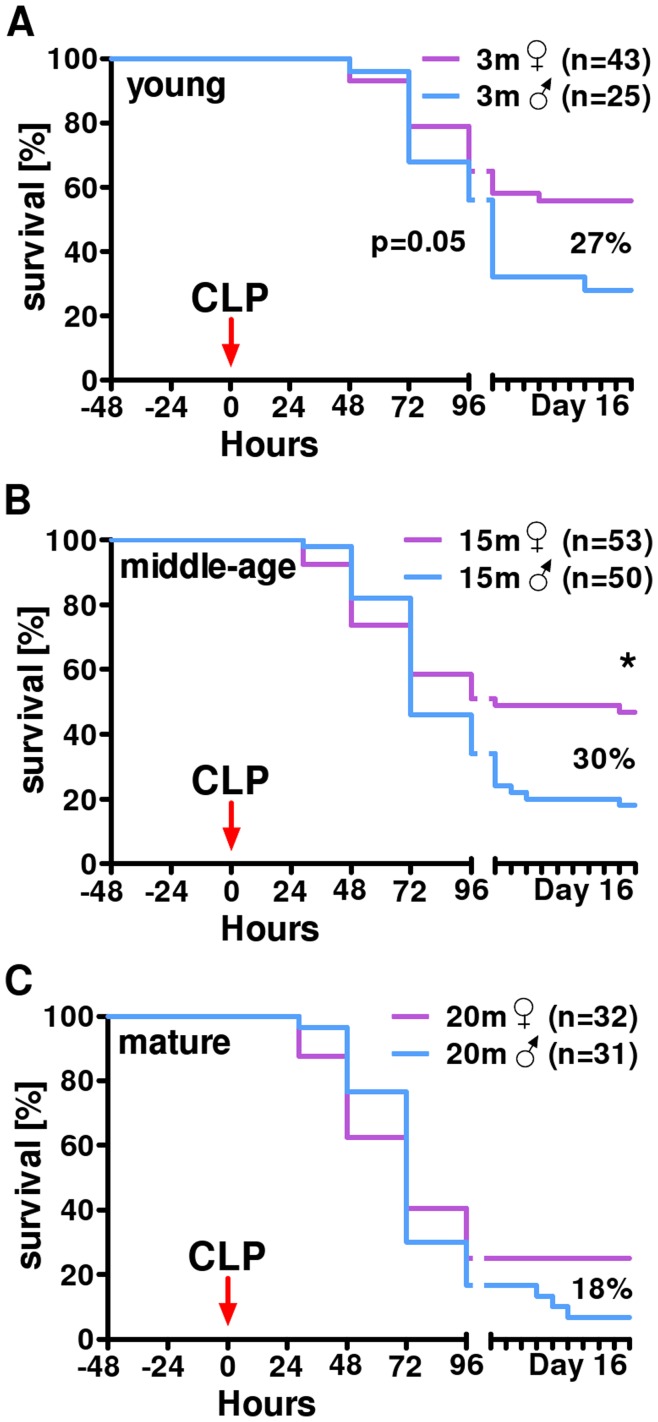
Survival in post-traumatic sepsis. 16-day mortality in 3 (A), 15 (B) and 20 month (C) old female and male mice subjected to trauma/hemorrhage followed by CLP sepsis. 3m♀ n = 43, 15m♀ n = 53, 20m♀ n = 32, 3m♂ n = 25, 15m♂ n = 50, 20m♂ n = 31.

**Figure 3:**
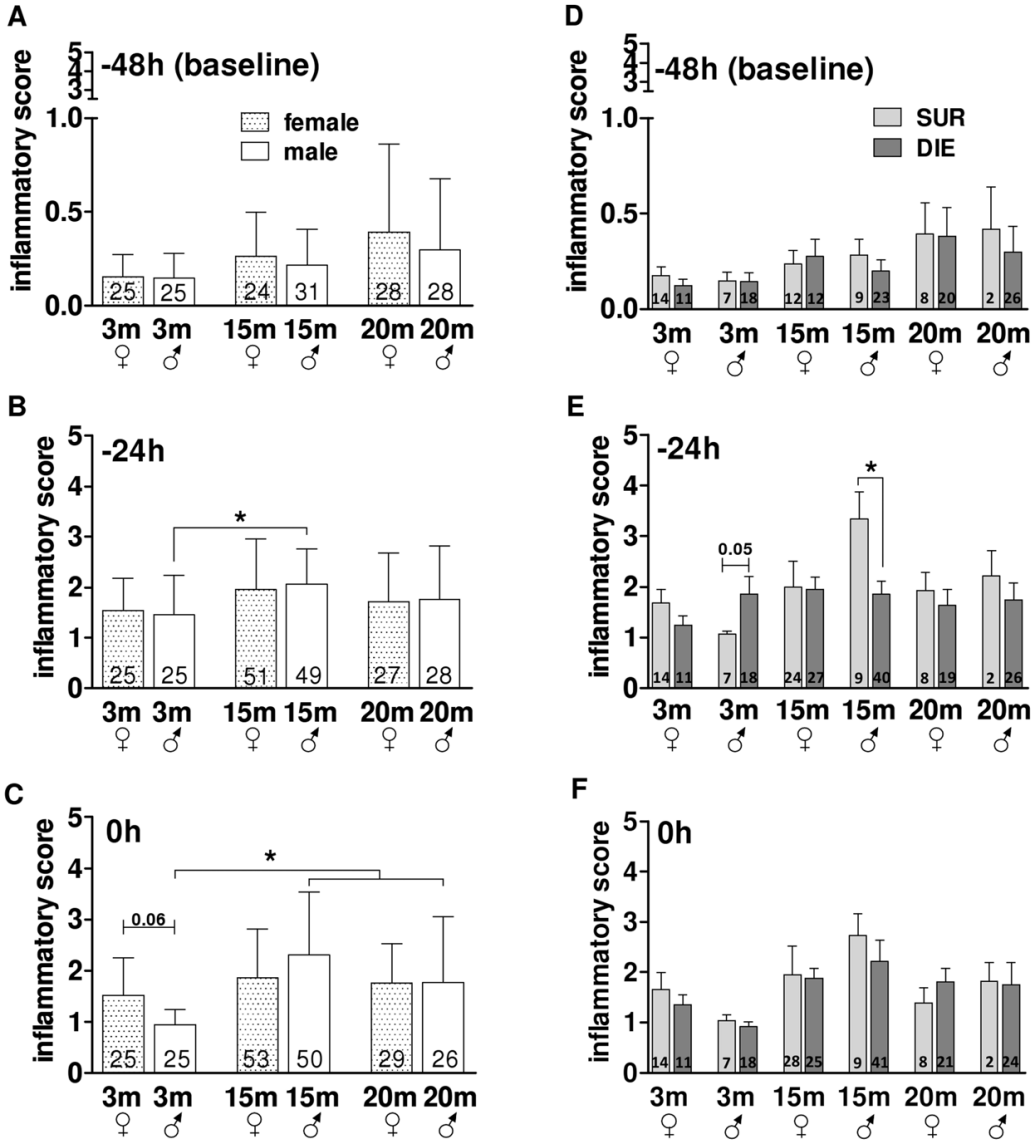
Pre-CLP Phase – the Comparison of the Composite Inflammatory Score in different age/gender groups. A–C. Comparison of composite inflammatory cytokine release score in 3, 15 and 20 month old female and male mice at −48 h, −24 h, 0 h prior CLP. D–E. Outcome based comparison of inflammatory cytokine release in 3, 15 and 20 month old female and male mice at −48 h, −24 h, 0 h prior CLP. SUR = alive on day 16, DIE = died until day 16 post-TH. Data presented as mean+SD. The number of animals/group listed in each bar. *p<0.05.

Evidence from available pre-and clinical studies are conflicting, hence the factual influence of age/gender upon early post-traumatic/hemorrhagic responses remains elusive. To examine global and outcome-dependent inflammatory responses after TH, we combined all cytokines and organ function parameters measured post-TH into general composite scores (by normalizing all individual cytokine and organ function parameter values - see statistical section and Fig. S1). Overall, trauma/hemorrhage was sublethal (Fig. 2) and induced a similar increase of the general inflammatory response (between 4.5-10-fold compared to baseline) across all groups (Fig. 3A–C). The only age-related difference occurred in males: at −24 h, the composite inflammatory response score in middle-age mice was approx. 1.5-fold higher compared to young (but not mature) animals (p<0.05) (Fig. 3B). At 0 h (i.e. immediately prior to CLP), the inflammatory score in the young group was on average 50% lower compared to all remaining males (and females) (Fig.3C). Regardless of age and gender, the peak of post-TH inflammatory activation at −24 h was consistently lower (at least 2-fold) compared to the peak of post-CLP activation at 24 h (Fig. S2).

Given that no apparent changes were detected in the above comparisons, in the next step we aimed to establish whether post-TH inflammatory responses in different age/gender groups vary contingent on post-septic outcomes. To achieve that, we additionally separated TH mice into the DIE and SUR subgroups (based on the post-CLP outcome; see the study design section). Composite inflammatory response scores were virtually identical between all SUR vs. DIE pairs, regardless of age and gender (Fig. 3A–E). The only transient difference occurred in male mice at −24 h: the inflammatory response score in middle-age SUR males was approx. 2-fold higher compared to middle-age DIE males and a similar trend was observed in young males (p = 0.05) (Fig. 3D).

#### Differential outcome-dependent release of IL-5, MIP-1α and IFN-γ in middle-age males

**Figure 4:**
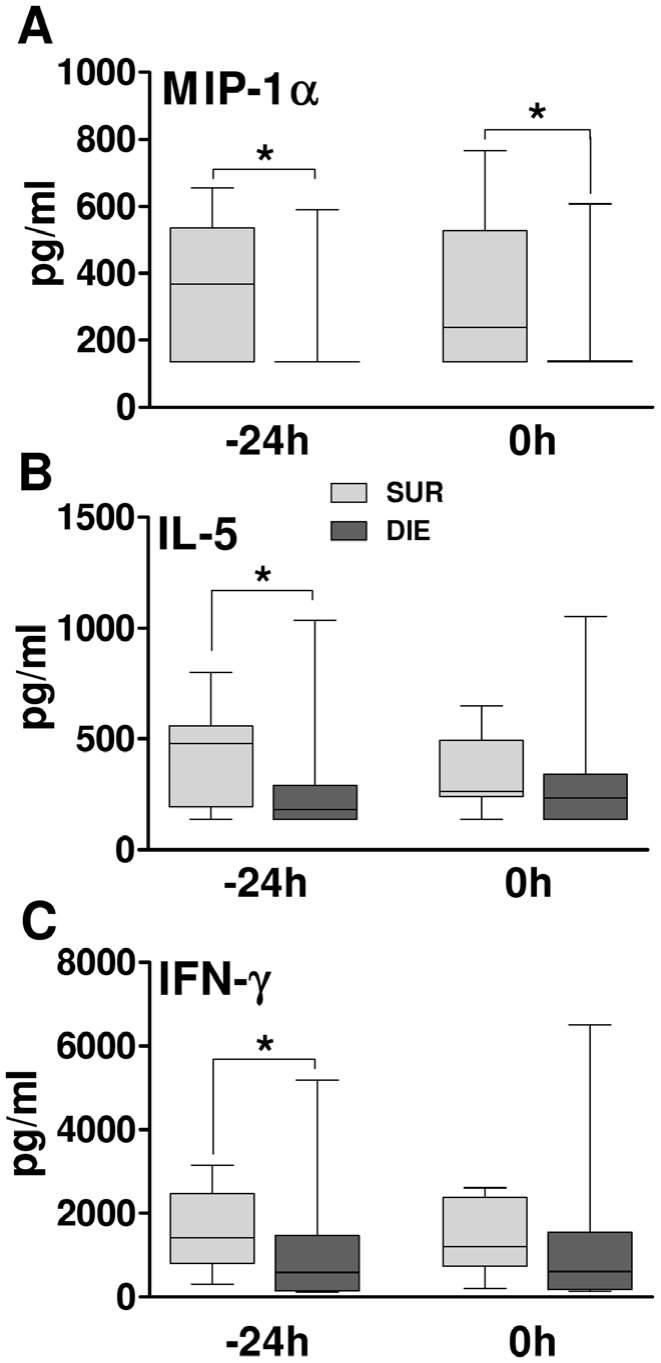
Pre-CLP phase – Comparison of outcome differences in individual selected cytokines. A–C. Outcome based comparison of plasma levels of circulating MIP-1α, IL-5 and IFN-γ in 15 month male mice at −24 h and 0 h prior CLP. SUR = alive on day 16, DIE = died until day 16. −24 h: SUR n = 9, DIE n = 40 and 0 h SUR n = 9, DIE n = 42. Data presented as box and whiskers (Min, IQR 2, Median, IQR 3, Max). *p<0.0.

Given the above DIE vs. SUR disparity in young and middle-age males, we wanted to establish whether any particular mediator(s) was responsible for this difference or this was due to small but consistent changes in all examined cytokines. Thus, we examined all cytokines in young and middle-age group to identify/assess the magnitude of DIE vs. SUR differences in each individual mediator separately. The most relevant SUR vs. DIE differences were observed in middle-age males at −24 h (Fig. 4): SUR IFN-γ, IL-5, and MIP-1α were higher by approx. 40%, 42% and 50% compared to DIE animals. Only for MIP-1α this difference persisted also at 0 h (p<0.05) (Fig.4B).

We then tested whether the magnitude of recorded post-TH changes between SUR and DIE mice show predictive potential for early post-CLP outcome. All three cytokines displayed a moderate outcome prediction potential at −24 h: IL-5 AUC = 0.74, for IFN-γ AUC = 0.73, and for MIP-1α AUC = 0.63 (and 0.73 at 0 h). No further outcome-dependent differences in individual cytokines were identified (data not shown).

#### Age and gender did not modify the organ dysfunction induced by trauma/hemorrhage

The magnitude of deregulation in functioning of vital organs after a tissue injury/hemorrhage, even when fully survivable, may greatly vary with age and gender. In this part of the study, we set out to assess to what extent age/gender can modulate organ function after the TH hit. Similar to cytokine response data above, separate composite organ dysfunction scores for each age/gender group were generated (see study design section and Fig. S1).

**Figure 5:**
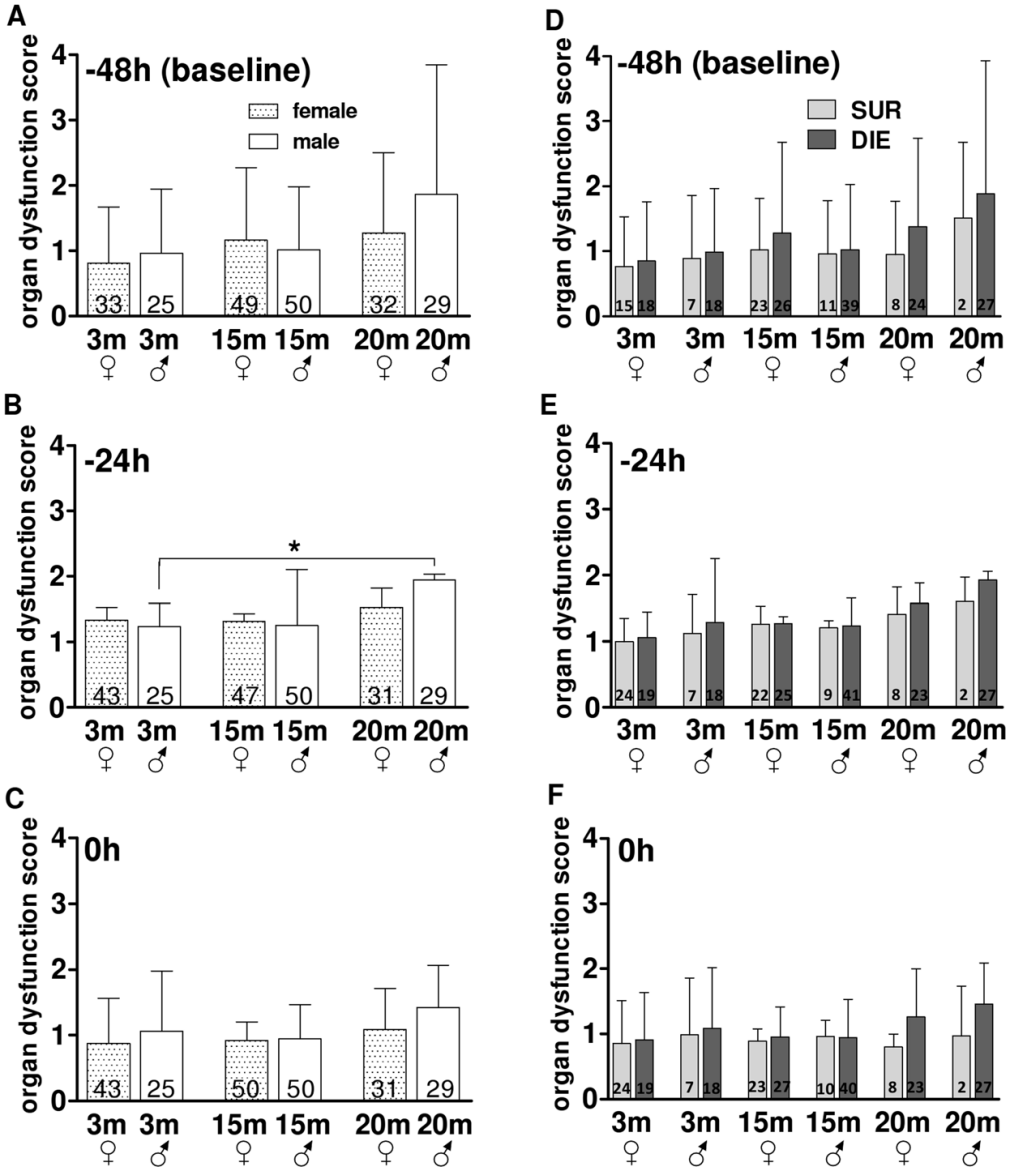
Pre-CLP Phase – the Comparison of the Composite Organ Dysfunction Score in different age/gender groups. A–C. Comparison of organ dysfunction score changes in 3, 15 and 20 month old female and male mice at −48 h, −24 h, 0 h prior CLP. D–E. Outcome based comparison of organ dysfunction score changes in 3, 15 and 20 month old female and male mice at −48 h, −24 h, 0 h prior CLP. SUR = alive on day 16, DIE = died until day 16 post-TH. Data presented as mean+SD. Dotted line represents organ dysfunction score normal values. The number of animals/group listed in each bar. *p<0.05.

Overall, TH induced only a mild increase of organ dysfunction score and this effect was fully independent of age and gender. Only a 1.6-fold elevation of organ dysfunction score was observed in mature males (vs. young) but this difference, although significant, was short-lived (Fig.5 B, C). Regardless of age and gender, the peak of post-TH organ dysfunction at −24 h was consistently lower compared to the peak of post-CLP dysfunction at 48 h (Fig. S3).

As in case of circulating cytokines, we also hypothesized that potential post-TH changes in organ dysfunction may depend on subsequent post-CLP outcomes. Surprisingly, retrospective separation of mice based on post-CLP outcome did not reveal any differences among the increases of composite organ dysfunction scores in the studied age and/or gender groups (Fig.5 D–F).

#### Age and gender did not modulate hypoglycemia and blood counts induced by trauma/hemorrhage

Decrease of blood glucose, as a measure of imbalanced sugar metabolism, and reduction in circulating Hb concentration and RBC counts, are inherent stress/blood loss symptoms of any post-TH challenge. We, therefore, analyzed whether age/gender meaningfully modulated these and other parameters (e.g. neutrophil and lymphocyte counts) in tested groups. In line with the organ dysfunction score comparison, the post-TH decrease of plasma glucose was similar in all mice (Fig. S4).

Additionally, the TH hit caused a similar reduction in the red blood cell count and hemoglobin concentration (by approx. 30%) that was independent of age, gender and outcome. The magnitude of this decrease remained virtually unchanged until 0 h (CLP). Also the slight post-TH thrombocytopenia was similar in all groups. As above (Fig. 3 and 5), we divided mice into DIE and SUR cohorts based on post-CLP outcome, and re-analyzed the data. In young males, circulating neutrophil and lymphocyte counts were consistently increased (by approx. 30%) in SUR compared to the DIE group at −24 h and 0 h. The opposite was true for neutrophil in middle-age females: DIE counts were approx. 40% higher (p<0.05) than SUR counts at −24 h (Figs. S5, S6, S7, S8, S9, S10).

### Post-CLP Phase

#### Increasing age and male gender predispose for the worse survival after post-traumatic sepsis

**Figure 6:**
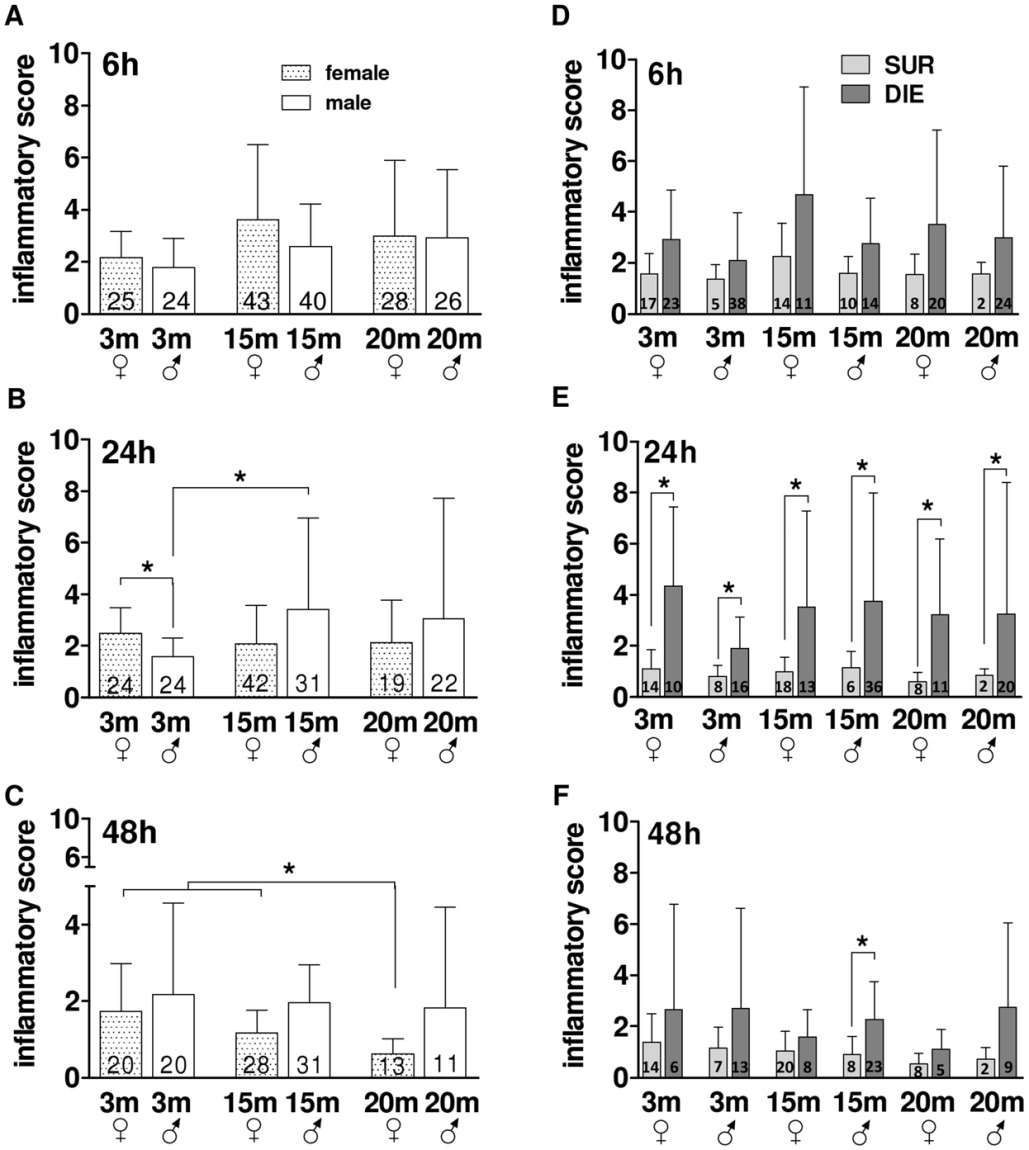
Post-CLP Phase – the Comparison of the Composite Inflammatory Score in different age/gender groups. A–C. Comparison of global inflammatory cytokine release in 3, 15 and 20 month old female and male mice at 6 h, 24 h and 48 h post-CLP. D–E. Outcome based comparison of inflammatory cytokine release in 3, 15 and 20 month old female and male mice at at 6 h, 24 h and 48 h. SUR = alive on day 16, DIE = died until day 16 post-TH. Data presented as mean+SD. The number of animals/group listed in each bar. *p<0.05.

**Figure 7:**
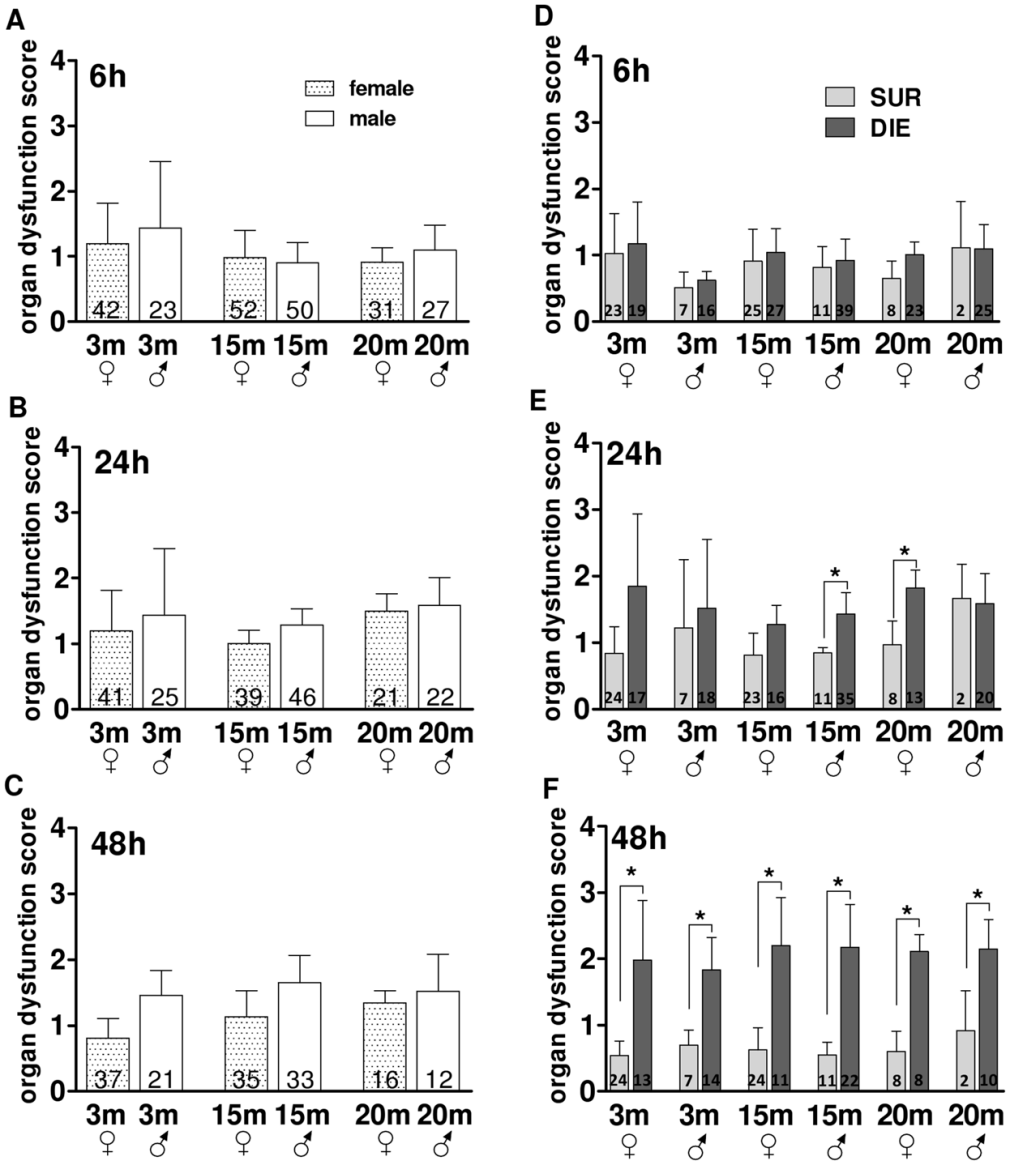
Post-CLP phase – the Comparison of the Composite Organ Dysfunction Score in different age/gender groups. A–C. Comparison of organ dysfunction score changes in 3, 15 and 20 month old female and male mice at 6 h, 24 h and 48 h post-CLP. D–E. Outcome based comparison organ dysfunction score changes in 3, 15 and 20 month old female and male mice at at 6 h, 24 h and 48 h. SUR = alive on day 16, DIE = died until day 16 post-TH. Data presented as mean+SD. Dotted line represents organ dysfunction score normal values. The number of animals/group listed in each bar. *p<0.05.

While the correlation between geriatric age and exacerbated mortality in patients suffering from (post-traumatic) sepsis is indisputable, the exact turning point of the increased, age-dependent susceptibility remains unclear. The ambiguity is far greater regarding the true role of gender in post-septic outcomes. This is why we attempted to determine effects of selected age and gender groups on survival in post-traumatic sepsis (Fig. 2), and simultaneously compare their inflammatory and organ function responses (subsequent Figs. 6–7).

All outbred CD-1 mice were subjected to TH-CLP and followed for 14 days (study design scheme - Fig.1). Given that the TH hit was sub-lethal, first deaths occurred at 6 h post-CLP onward (Fig. 2). A general decline in 14-day survival of both female and male mice was observed with increasing age. Independent of age, females had consistently better survival compared to males in corresponding age groups (3 m group: 55% vs. 28%; 15 m: 46% vs. 18% and 20 m: 25% vs. 7%). Of note, a relatively consistent magnitude of survival difference between females and males among different age group was observed: 27% in young, 28% in middle-age and 18% in mature (Fig. 2).

#### Estrus cycle status was not related to mortality in post-traumatic sepsis

Numerous studies implicated estrogen as the primary factor behind the survival advantage in septic females of reproductive age over age-matched male subjects [37]. As oscillation of circulating estrogen is tightly correlated to ovulation, we aimed to investigate whether the estrus cycle status was related to survival after TH-CLP. For comparisons, we collected vaginal smears from a selected group of young and middle-age female mice at the onset of the experiment.

In general, distribution of estrus cycle phases showed a higher incidence of estrus in young versus middle-age females. However, death and/or survival were not related to any predominating phase of estrus cycle in neither of the age groups we evaluated in this study (Table 1).

**Table 1:** Distribution of estrus cycle phases prior to TH-CLP.

Estrus cycle phase	3m ♀ SUR	3m♀ DIE	15m♀ SUR	15m♀ DIE	20m♀ SUR	20m♀ DIE
Total n	11 (%)	8 (%)	12 (%)	16 (%)	1 (%)	12 (%)
**Proestrus**	1 (9)	–	–	1 (6)	1	–
**Estrus**	4 (36)	3 (37.5)	1 (8)	2 (12.5)	–	1 (8)
**Metestrus**	2 (19)	2 (25)	6 (50)	7 (44)	–	3 (25)
**Diestrus**	4 (36)	3 (37.5)	5 (42)	6 (37.5)	–	8 (67)

#### Age and gender-related changes in the global inflammatory response induced by post-traumatic sepsis

Early sepsis is characterized by a robust release of multiple pro-and anti-inflammatory cytokines into the blood, and a number of studies demonstrated that magnitude of this release may markedly differ between genders and groups at opposite ends of the age spectrum. We wanted to re-examine this facet, yet in the context of an age/gender-dependent, potentially differential, influence of the post-TH inflammatory activation. The most consistent gender effect was observed in middle-age and mature females (Fig. 6A–C): compared to respective male groups, females showed a generally depressed cytokine response. This difference, already present at 24 h, was the most pronounced between mature males and females at 48 h (approx. 65%). Furthermore, cytokine response was much lower in mature females (by at least 1.9-fold) compared to middle-age and young females at 48 h (p<0.05).

Except for young males, whose composite inflammatory response score was decreased compared to young females (and middle-age males) at 24 h (Fig. 6B), the overall inflammatory response among remaining male groups was virtually identical at all other time points (Fig. 6C).

It has been demonstrated that acute humoral inflammatory release of several cytokines (e.g. IL-6, MIP-2, IL-10) tightly correlates with early septic outcomes. In the next analysis, we aimed to examine potential outcome-related differences in the global cytokine signature among age and gender groups. To achieve this, all age/gender groups were, based on post-septic outcome, retrospectively separated into DIE and SUR subgroups (Fig. 6D–F). The stratification revealed an enhanced inflammatory response in all DIE mice from 6 h onward. The strongest separation was recorded at 24 h post-CLP (DIE approx. 3.7-fold higher vs. SUR) (Fig. 6E): although it was relatively weaker in young males, the magnitude of the SUR vs. DIE separation was similar among remaining age/gender groups.

#### Age and gender did not modify organ dysfunction induced by post-traumatic sepsis

Rapid and excessive inflammatory response occurring in early sepsis is considered to be co-responsible for induction of progressive organ dysfunction and subsequent MODS. Since deregulation of organ function homeostasis may be differentially modulated by both age and gender, we aimed to characterize their influence upon organ status changes in the early phase of post-traumatic sepsis. Not surprisingly, a rapid increase of the composite organ dysfunction score occurred immediately after CLP and remained elevated until 48 h (Fig. 7A–C). However, this elevation was identical across all age and gender groups.

Next, we tested whether age and/or gender had meaningful impact upon outcome-dependent (i.e. SUR vs. DIE) deregulation of organ function in the first days of post-traumatic sepsis. In general, retrospective stratification revealed an increasing separation of DIE (rising faster) vs. SUR organ dysfunction scores from 24 h onward. This was first observed only in middle-age male and mature female DIE mice (higher by 40% and 50% vs. SUR, p<0.05) (Fig. 7D,E). At 48 h, all DIE mice displayed the peak increase in organ dysfunction score compared to SUR. Surprisingly, these outcome-based differences were similar (approx. 3-fold) and independent of age and gender (Fig.7F).

#### Age and gender-related changes in the blood counts induced by post-traumatic sepsis

As both trauma/hemorrhage and sepsis alone exert strong changes on the profile of circulating red and white blood cells, in the final analysis, we investigated how secondary sepsis affected differential cell counts in the context of the post-CLP outcome.

Overall, post-TH-CLP trajectories of red blood cell and platelet counts as well as hemoglobin concentration were very similar across all age/gender groups and independent of outcomes. A number of transient changes were observed in leukocyte subpopulations (Figs. S11, S12, S13, S14, S15, 16). At 24 h post-CLP, young female SUR mice had a decreased number of lymphocytes (by approx. 2.5-fold) compared to the young female DIE group (Fig. S11). This reversed at 72 h: both young female and male SUR mice showed a slight recovery of lymphocyte counts compared to DIE animals (increased by approx. 2 and 4.5-fold). In middle-age SUR males, neutrophil counts were higher compared to middle-age DIE males at 48 h and 72 h (by approx. 40% and 70%, p<0.05) (Figs. S11–16).

## Discussion

The current investigation of the age/gender-related impact upon the responses in post-traumatic sepsis revealed three main findings: 1) post-septic deaths could not be accurately predicted by the trauma/hemorrhage-induced responses (i.e. measured prior to the sepsis onset), 2) the gender/age-related differences in post-CLP survival were not reflected by changes in the magnitude of the inflammatory activation and/or organ dysfunction, and 3) the magnitude of outcome-dependent (i.e. DIE vs. SUR) changes in the inflammatory/organ function response during the post-septic period were virtually identical across all age/gender groups. Summary of the main findings of the study is listed in Table 2.

**Table 2:** Summary of the main findings of the study.

**Post-TH/Pre-CLP:**
• Inflammatory/organ function responses were not overtly modulated by gender/age
• Inflammatory Response Score was weakest in young males
• Prediction of acute septic outcomes based on pre-CLP cytokine release lacked accuracy
**Post-CLP:**
• Increasing age corresponded with increasing mortality
• Survival rate in females was higher than in age-matched males
• Outcomes were not explicitly associated to any specific estrus cycle phase
• Age/gender survival differences were not reflected by changes in global Inflammatory/Organ Dysfunction Scores
• The magnitude of SUR vs. DIE differences in global Inflammatory/Organ Dysfunction Score was similar and independent of age/gender

To date, virtually all experimental [9], [10], [38] and clinical [14], [15], [39], [40] studies investigating impact of age and/or gender on trauma/hemorrhage and sepsis, were limited either to only selected immuno-inflammatory parameters in survival studies (i.e. maximally 9 parameters) and/or by interval sacrificing of animals for high throughput screenings. To characterize the evolution of age/gender-dependent immune and organ function responses in an uninterrupted fashion without reducing the number of biomarkers, we integrated the post-traumatic sepsis model with the daily facial vein sampling technique [29] allowing us to screen the total of 20 parameters over the period of 7 days. Furthermore, we utilized a traumatic insult of medium severity (i.e. femur fracture with soft tissue injury) that was paired with an acute (sublethal) blood loss. Such a combination was prompted by the report of Wichmann et al. who demonstrated that bone fracture coupled with hemorrhagic shock is more clinically relevant (e.g. compared to the widely used laparatomy/hemorrhage combination) as it exacerbates immune depression induced by hemorrhage alone [41].

The humoral immuno-inflammatory and organ function equilibrium in any inflammatory syndrome is maintained by a complex interplay among many circulating mediators and rarely a single decisive biomarker is responsible for the ultimate outcome. We, therefore, generated two composite scores (representing the two aforementioned systems) to gain insight into the global response patterns in different age and gender groups rather than investigate the entire palette of individual biomarkers separately. For the age selection, we used the classification by Turnbull et al., as a general guideline: the study defined 3 month old C57BL/6 mice as comparable to a human age of 10 years, while 15 and 20 months old mice representing the human age of 50 and 65 years [42]. Specifically, the choice of the two latter age groups was made to encompass the age-range at which patients start to develop/succumb to sepsis most frequently [18], [43], yet without displaying an evident senescent phenotype. In the CD-1 strain, the transition from 15 to 20 month of age constitutes the beginning of a steep deterioration phase in male/female mortality, but it precedes the most accelerated mortality rate occurring in the geriatric age [44]. Hence, the 15 and 20 month old mice used in our study approximately represent physiology of middle-age and mature humans.

The first finding of our study is uncontroversial - both age and gender markedly influenced survival after TH-CLP. While the incidence of deaths gradually rose with the increasing age (regardless of gender), female mice demonstrated a consistently better survival than the age-matched males. This effect agrees with a number of earlier studies. For example, Sperry et al., 2008 [12] associated female gender with a significantly lower mortality and reduced rate of multiple organ dysfunctions after trauma and blood loss – both independently of age and reproductive status of female patients. It has been also demonstrated that young female mice in proestrus tolerate hemorrhage and sepsis better than males of the same age [11], [38], [45]. In the context of the absence of gender-dependent effects upon mortality in the mature group (Fig. 2C), a similar finding was reported by Mees et al. (2007) [46] who showed no significant survival difference between 18–19 month old female and male mice subjected to a milder two-hit model (hemorrhage-only followed by sepsis).

What was more interesting in the overall context of post-traumatic outcomes is that we were not able to associate post TH-CLP death/survival, regardless of age, to any apparent shift in the incidence of the estrus cycle phases. This could be either a strain-specific phenomenon or more likely, the 1^st^ hit might have interrupted the sexual cycle so that the estrus cycle status in all females was similar immediately prior to subjecting them to the 2^nd^ hit (CLP). In addition, an assessment of the hormonal profile we performed in healthy mice in the same age groups (3, 15 and 20 months) did not reveal an evident reduction of estrogen with increasing age (data not shown). Remarkably, re-assessment of healthy 20 month old females four months later demonstrated that their average estrogen concentration matched the one recorded in 20 month old females despite no incidences of estrus phase by vaginal smears. Additionally, preliminary data of the same study implied that vaginal smears utterly fail to reliably pinpoint pre-estrus/estrus peaks in circulating estrogen in ovulating mice (data not shown).

The most remarkable finding of our study, however, is that neither gender nor age evidently affected the global immuno-inflammatory and organ function response induced by either TH alone or TH-CLP combined. Although some infrequent statistically significant differences among groups were recorded, we were not able to dissect any clear and consistent gender/age-shifts within the scope of the studied endpoints. A late (at 0 h) and transient post-TH change was observed in young males: their overall cytokine release (but not organ function) was markedly weaker compared to (majority of) the remaining groups (Fig.3C). As the existing literature is scarce, this observation is currently unsubstantiated. The only, relatively more convincing, trend was observed after the 2^nd^ hit (CLP): the magnitude of the inflammatory response gradually decreased with age (Fig. 6C and F), yet this was true only in females and recorded at 48 h but not earlier. This finding appears to contrast a number of studies showing that in old mice (compared to the young counterparts suffering from an equivalent insult), exacerbated mortality was accompanied by increased systemic and local inflammation after sepsis [42], [47], [48]. Clearly, the smaller-than-expected impact of inflammatory and organ function compartments upon the age/gender-dependent mortality within the studied groups require solid verification. We, however, allow a speculation that the observed demographic increase in vulnerability to sepsis (in the population at the approx. age of 60), must not be immediately related to any major shift in the immuno-inflammatory compartment, at least on the humoral level. In other words, the age-dependent immuno-inflammatory effects on incidence/outcome in sepsis may play a decisive role only at the very late, geriatric age. The comparison of outcome-dependent profiles in the next two paragraphs lends further support to the above speculations.

One of the most challenging aspects of sepsis as the secondary complication is that it is highly unpredictable regarding both its occurrence and outcome. To predict impeding complication and/or predict outcome in an already on-going secondary sepsis would be a milestone achievement. Disappointingly, numerous studies searching for biomarkers able to fulfill the above goals, have produced unsatisfying results [49]–[51]. Thanks to the protracted monitoring and retrospective DIE/SUR stratification, we approached the milestone of predicting outcomes in secondary sepsis in an entirely new context: i.e., whether it is feasible to accurately prognosticate early deaths after the 2^nd^ hit sepsis based on the TH-responses developing during the preceding 48 h. Regrettably, our study failed to provide any solid outcome-predisposing factors related to either age or gender. The only exception was the middle-age male group, in which we observed a marked outcome-dependent difference in the inflammatory response, with MIP-1α, IL-5 and IFN-γ responsible for the separation (Fig. 4). Although the ROC analysis did not confirm a strong outcome-predictive capacity of these cytokines, the recorded changes should not be easily dismissed. Given that all animal subgroups were relatively homogenous (i.e. no comorbidities present, similar immuno-inflammatory status prior to the 1^st^ hit) and subjected to CLP (of the same severity) at the same time, the experimental design is very conservative. These relatively moderate outcome-based cytokine differences may become much more pronounced in more heterogeneous clinical cohorts and should be monitored.

Finally, using the same retrospective approach, we aimed to gain a deeper insight into the lethal mechanisms of secondary sepsis: we compared the varying survival rates to the magnitude of changes in the global inflammatory response and organ dysfunction scores between SUR and DIE mice across all post-CLP groups. Since an excessive release of immuno-inflammatory biomarkers is considered the key contributor to post-septic MODS and subsequent mortality [52], it was intuitive to expect that the changes in the inflammatory response and/or organ dysfunction scores should parallel the age-dependent alterations in mortality. Surprisingly, neither the changes in the inflammatory response nor in the organ dysfunction score reflected the observed differences in survival. It is evident that the lack of correlation between those two elements (i.e. diminishing survival and changes in inflammatory response/organ dysfunction) was not due to an inappropriate calibration of the TH-CLP severity: the separation of the DIE/SUR responses in both compartments was evident in all mice (especially at the peak of their activation). Yet, the magnitude of differences between SUR and DIE mice remained always identical across virtually all age/gender groups.

### Conclusion

By combining a relevant 2-hit TH-CLP model and low-volume daily blood sampling, this study for the first time provided a protracted and wide-ranging characterization of the evolution of the immuno-inflammatory responses and organ dysfunction in the course of post-traumatic sepsis. It additionally allowed a unique comparison of these response patterns across age, gender and septic outcomes (summarized in Table 2). Overall, the TH-CLP induced changes in the global immuno-inflammatory and organ dysfunction signatures were surprisingly uniform. This was especially striking after the outcome-based stratification, which revealed that the age/gender survival differences were not reflected by the portrayed response patterns between the corresponding groups. Whereas undeniably influential, the exact role of gender/age upon outcomes in (post-traumatic) sepsis remains undefined and invites further experimental and clinical scrutiny.

## Supporting Information

Figure S1 **Schematic of the Composite Inflammatory Score Calculation in the pre-CLP (A) and post-CLP (B) phase of post-traumatic sepsis.** Calculation of the Composite Scores served as a tool enabling general comparison of inflammatory responses (and organ dysfunction) among all studied age/gender groups. To effectively normalize single cytokine values in all individual mice a median cytokine value was selected and applied for normalization of all cytokine values to a uniform mathematical denominator. Normalization of each cytokine was performed separately using an individual median value for this specific cytokine. The normalizing median values were selected from the −24 h (pre-CLP) and 24 h (post-CLP) data sets given that at those time-points activation of inflammatory response was highest. Although this schematic uses an exemplary IL-1β cytokine, the identical step-by-step normalization protocol was employed for calculation of the Composite Organ Dysfunction Score. Final group comparisons were based on nine Composite Inflammatory Score mediators, and four Composite Organ Dysfunction score parameters (i.e. ALT; AST, urea and LDH).
(TIF)

Figure S2 **Comparison of the Composite Inflammatory Score in different age/gender groups across two phases of post-traumatic sepsis.** Separate score trajectories in 3 (A and C), 15 (B and D) and 20 (C and E) month old female and male mice at −48 h, −24 h, 0 h prior CLP, and 6 h, 24 h and 48 h post-CLP are provided. To enable an overview of inflammatory activation across the entire TH-CLP period, the same median value (i.e. from the 24 h data set) was used for normalization of each cytokine in both pre-and post-CLP phases. Data presented as mean+SEM. The number of animals/group per time-point is identical with n listed in Figs. 3 and 6. No statistical comparisons among age/gender groups are provided here as they are detailed in the respective Figs. 3 and 6.
(TIF)

Figure S3 **Comparison of the Composite Organ Dysfunction Score in different age/gender groups across two phases of post-traumatic sepsis.** Separate score trajectories in 3 (A and C), 15 (B and D) and 20 (C and E) month old female and male mice at −48 h, −24 h, 0 h prior CLP, and 6 h, 24 h and 48 h post-CLP are provided. To enable an overview of organ dysfunction across the entire TH-CLP period, the same median value (i.e. from the 24 h data set) was used for normalization of each cytokine in both pre-and post-CLP phases. Data presented as mean+SEM. The number of animals/group per time-point is identical with n listed in Figs. 3 and 6. No statistical comparisons among age/gender groups are provided here as they are detailed in the respective Figs. 5 and 7.
(TIF)

Figure S4 **Pre-CLP phase: plasma glucose levels in different age/gender groups.** A–F. Glucose plasma levels at −48 h, −24 h and 0 h prior to CLP. SUR = alive on day 16, DIE = died until day 16 post-TH. Data presented as mean+SD. Dotted line represents normal values. In 3 m♀ SUR n≥15, in DIE n≥18 at all time points. In 3 m♂ SUR n = 7, in DIE n = 18 at all time points. In 15 m♀ SUR n≥23, in DIE n = 51 at all time points. In 15 m♂ SUR n≥8, in DIE n≥41 at all time points. In 20 m♀ n = 8, in DIE n = 23 at all time points. In 20 m♂ SUR n = 2, in DIE n = 23 at all time points.*p<0.05
(TIF)

Figure S5 **Pre-CLP phase: complete cell count in 3 month old female mice.** A–C. Levels of circulating red blood cells (RBC), hemoglobin (Hb) and platelets (PLT) at −48 h, −24 h and 0 h prior CLP. D+E. Circulating neutrophils (NEU) and lymphocytes (LYM) at −48 h, −24 h and 0 h prior to CLP. SUR = alive on day 16, DIE = died until day 16 post-TH. Data presented as box and whiskers (Min, IQR 2, Median, IQR 3, Max). Dotted line represents normal values. In SUR n≥18, in DIE n≥22 at all time points.
(TIF)

Figure S6 **Pre-CLP phase: complete cell count in 3 month old male mice.** A–C. Levels of circulating red blood cells (RBC), hemoglobin (Hb) and platelets (PLT) at −48 h, −24 h and 0 h prior CLP. D+E. Circulating neutrophils (NEU) and lymphocytes (LYM) at −48 h, −24 h and 0 h prior to CLP. SUR = alive on day 16, DIE = died until day 16 post-TH. Data presented as box and whiskers (Min, IQR 2, Median, IQR 3, Max). Dotted line represents normal values. In SUR n = 7, in DIE n = 18 at all time points, *p<0.05.
(TIF)

Figure S7 **Pre-CLP phase: complete cell count in 15 month old female mice.** A–C. Levels of circulating red blood cells (RBC), hemoglobin (Hb) and platelets (PLT) at −48 h, −24 h and 0 h prior CLP. D+E. Circulating neutrophils (NEU) and lymphocytes (LYM) at −48 h, −24 h and 0 h prior to CLP. SUR = alive on day 16, DIE = died until day 16 post-TH. Data presented as box and whiskers (Min, IQR 2, Median, IQR 3, Max). Dotted line represents normal values. In SUR n≥20, in DIE n = 24 at all time points, *p<0.05.
(TIF)

Figure S8 **Pre-CLP phase: complete cell count in 15 month old male mice.** A–C. Levels of circulating red blood cells (RBC), hemoglobin (Hb) and platelets (PLT) at −48 h, −24 h and 0 h prior CLP. D+E. Circulating neutrophils (NEU) and lymphocytes (LYM) at −48 h, −24 h and 0 h prior to CLP. SUR = alive on day 16, DIE = died until day 16 post-TH. Data presented as box and whiskers (Min, IQR 2, Median, IQR 3, Max). Dotted line represents normal values. In SUR n = 9, in DIE n = at least 37 at all time points.
(TIF)

Figure S9 **Pre-CLP phase: complete cell count in 20 month old female mice.** A–C. Levels of circulating red blood cells (RBC), hemoglobin (Hb) and platelets (PLT) at −48 h, −24 h and 0 h prior CLP. D+E. Circulating neutrophils (NEU) and lymphocytes (LYM) at −48 h, −24 h and 0 h prior to CLP. SUR = alive on day 16, DIE = died until day 16 post-TH. Data presented as box and whiskers (Min, IQR 2, Median, IQR 3, Max). Dotted line represents normal values. In SUR n≥6, in DIE n≥21 at all time points.
(TIF)

Figure S10 **Pre-CLP phase: complete cell count in 20 month old male mice.** A–C. Levels of circulating red blood cells (RBC), hemoglobin (Hb) and platelets (PLT) at −48 h, −24 h and 0 h prior CLP. D+E. Circulating neutrophils (NEU) and lymphocytes (LYM) at −48 h, −24 h and 0 h prior to CLP. SUR = alive on day 16, DIE = died until day 16 post-TH. Data presented as box and whiskers (Min, IQR 2, Median, IQR 3, Max). Dotted line represents normal values. In SUR n = 2, in DIE n≥28 at all time points.
(TIF)

Figure S11 **Post-CLP phase: complete cell count in 3 month old female mice.** A–C. Levels of circulating red blood cells (RBC), hemoglobin (Hb) and platelets (PLT) at −48 h, −24 h and 0 h prior CLP. D+E. Circulating neutrophils (NEU) and lymphocytes (LYM) at −48 h, −24 h and 0 h prior to CLP. SUR = alive on day 16, DIE = died until day 16 post-TH. Data presented as mean+SEM. Dotted line represents normal values. In SUR n≥12 at all time points, in DIE at 6 h n = 11, at 24 h n = 10, at 48 h n = 5, and at 72 h n = 4. *p<0.05
(TIF)

Figure S12 **Post-CLP phase: complete cell count in 3 month old male mice.** A–C. Levels of circulating red blood cells (RBC), hemoglobin (Hb) and platelets (PLT) at −48 h, −24 h and 0 h prior CLP. D+E. Circulating neutrophils (NEU) and lymphocytes (LYM) at −48 h, −24 h and 0 h prior to CLP. SUR = alive on day 16, DIE = died until day 16 post-TH. Data presented as mean+SEM. Dotted line represents normal values. In SUR n≥7 at all time points, in DIE at 6 h n = 18, at 24 h n = 18, at 48 h n = 14, and at 72 h n = 9. *p<0.05.
(TIF)

Figure S13 **Post-CLP phase: complete cell count in 15 month old female mice.** A–C. Levels of circulating red blood cells (RBC), hemoglobin (Hb) and platelets (PLT) at −48 h, −24 h and 0 h prior CLP. D+E. Circulating neutrophils (NEU) and lymphocytes (LYM) at −48 h, −24 h and 0 h prior to CLP. SUR = alive on day 16, DIE = died until day 16 post-TH. Data presented as mean+SEM. Dotted line represents normal values. In SUR n≥17 at all time points, in DIE at 6 h n = 26, at 24 h n = 14, at 48 h n = 10, and at 72 h n = 4. *p<0.05
(TIF)

Figure S14 **Post-CLP phase: complete cell count in 15 month old male mice.** A–C. Levels of circulating red blood cells (RBC), hemoglobin (Hb) and platelets (PLT) at −48 h, −24 h and 0 h prior CLP. D+E. Circulating neutrophils (NEU) and lymphocytes (LYM) at −48 h, −24 h and 0 h prior to CLP. SUR = alive on day 16, DIE = died until day 16 post-TH. Data presented as mean+SEM. Dotted line represents normal values. In SUR n≥8 at all time points, in DIE at 6 h n = 40, at 24 h n = 39, at 48 h n = 22, and at 72 h n = 10. *p<0.05
(TIF)

Figure S15 **Post-CLP phase: complete cell count in 20 month old female mice.** A–C. Levels of circulating red blood cells (RBC), hemoglobin (Hb) and platelets (PLT) at −48 h, −24 h and 0 h prior CLP. D+E. Circulating neutrophils (NEU) and lymphocytes (LYM) at −48 h, −24 h and 0 h prior to CLP. SUR = alive on day 16, DIE = died until day 16 post-TH. Data presented as mean+SEM. Dotted line represents normal values. In SUR n≥8 at all time points, in DIE at 6 h n = 21, at 24 h n = 14, at 48 h n = 6.
(TIF)

Figure S16 **Post-CLP phase: complete cell count in 20 month old male mice.** A–C. Levels of circulating red blood cells (RBC), hemoglobin (Hb) and platelets (PLT) at −48 h, −24 h and 0 h prior CLP. D+E. Circulating neutrophils (NEU) and lymphocytes (LYM) at −48 h, −24 h and 0 h prior to CLP. SUR = alive on day 16, DIE = died until day 16 post-TH. Data presented as mean+SEM. Dotted line represents normal values. In SUR n = 2 at all time points, in DIE at 6 h n = 25, at 24 h n = 20, at 48 h n = 10, and at 72 h n = 2
(TIF)
