# Supplementary figures and images for: Relationship between Age/Gender-Induced Survival Changes and the Magnitude of Inflammatory Activation and Organ Dysfunction in Post-Traumatic Sepsis
Source: PLoS One. 2012 Dec 12;7(12):e51457. doi: 10.1371/journal.pone.0051457 (PMC3520804; doi:10.1371/journal.pone.0051457)

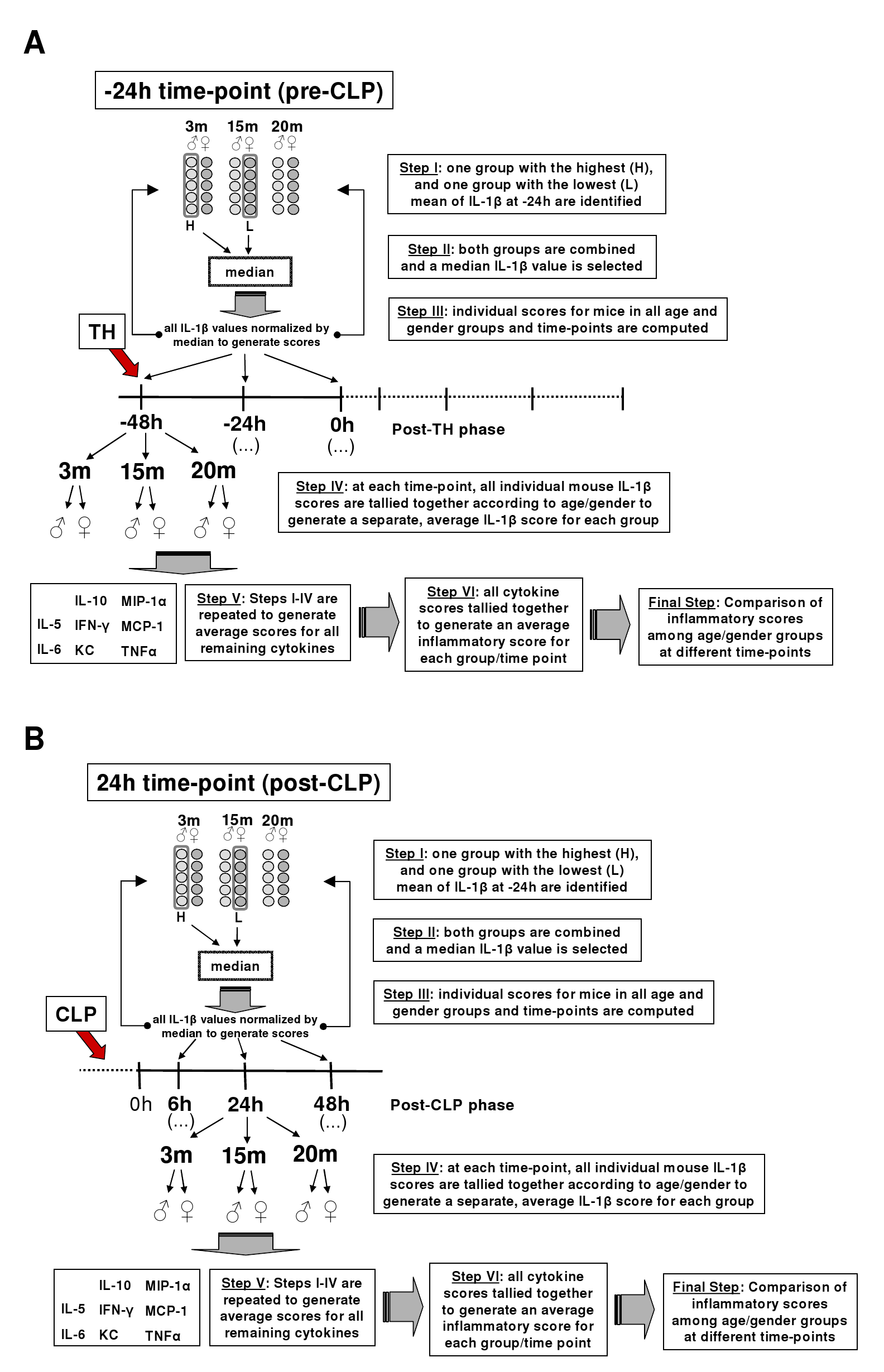

Supplement: Figure S1 — Schematic of the Composite Inflammatory Score Calculation in the pre-CLP (A) and post-CLP (B) phase of post-traumatic sepsis. Calculation of the Composite Scores served as a tool enabling general comparison of inflammatory responses (and organ dysfunction) among all studied age/gender groups. To effectively normalize single cytokine values in all individual mice a median cytokine value was selected and applied for normalization of all cytokine values to a uniform mathematical denominator. Normalization of each cytokine was performed separately using an individual median value for this specific cytokine. The normalizing median values were selected from the −24 h (pre-CLP) and 24 h (post-CLP) data sets given that at those time-points activation of inflammatory response was highest. Although this schematic uses an exemplary IL-1β cytokine, the identical step-by-step normalization protocol was employed for calculation of the Composite Organ Dysfunction Score. Final group comparisons were based on nine Composite Inflammatory Score mediators, and four Composite Organ Dysfunction score parameters (i.e. ALT; AST, urea and LDH). (TIF) [file pone.0051457.s001.tif]

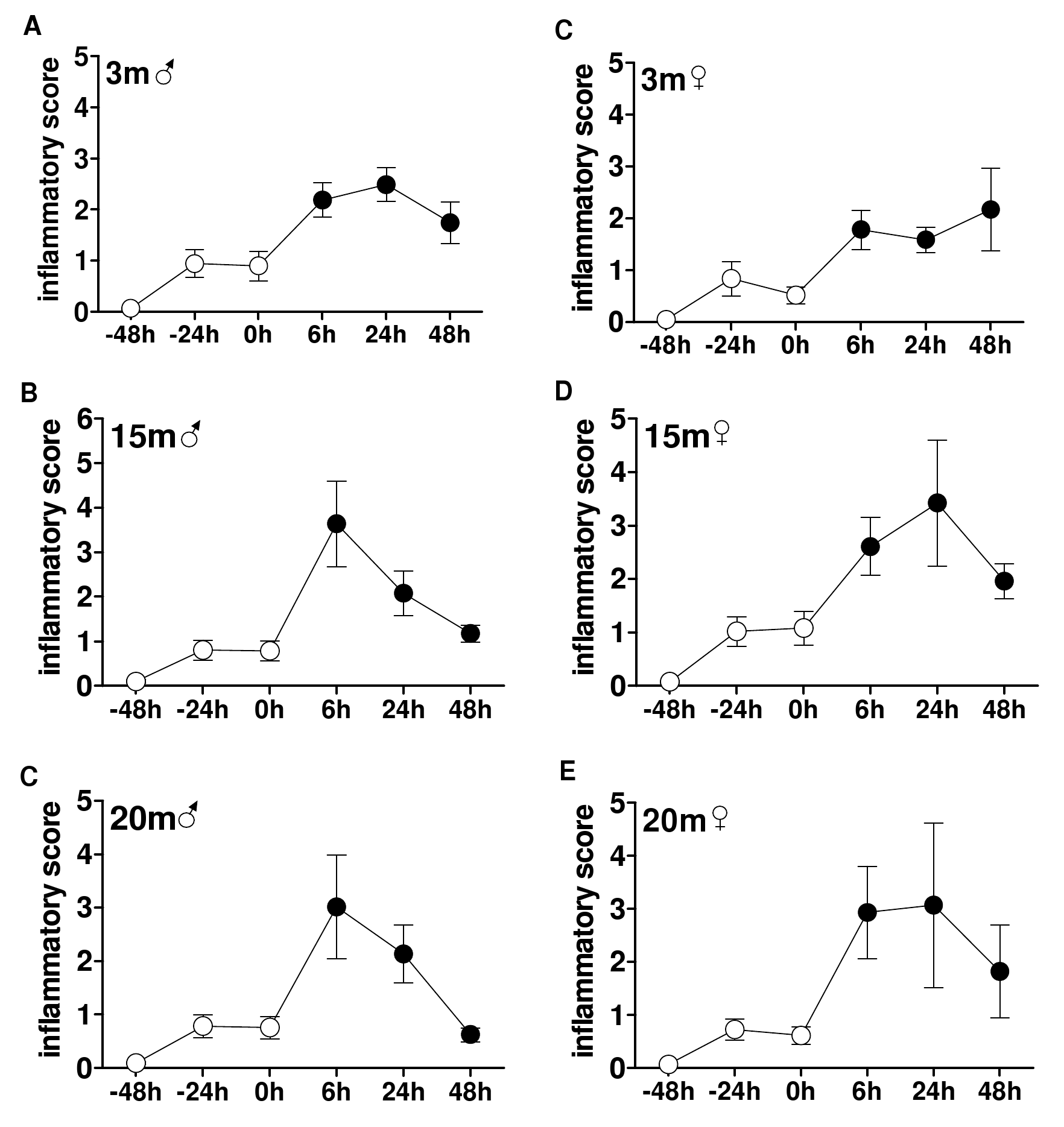

Supplement: Figure S2 — Comparison of the Composite Inflammatory Score in different age/gender groups across two phases of post-traumatic sepsis. Separate score trajectories in 3 (A and C), 15 (B and D) and 20 (C and E) month old female and male mice at −48 h, −24 h, 0 h prior CLP, and 6 h, 24 h and 48 h post-CLP are provided. To enable an overview of inflammatory activation across the entire TH-CLP period, the same median value (i.e. from the 24 h data set) was used for normalization of each cytokine in both pre-and post-CLP phases. Data presented as mean+SEM. The number of animals/group per time-point is identical with n listed in Figs. 3 and 6. No statistical comparisons among age/gender groups are provided here as they are detailed in the respective Figs. 3 and 6. (TIF) [file pone.0051457.s002.tif]

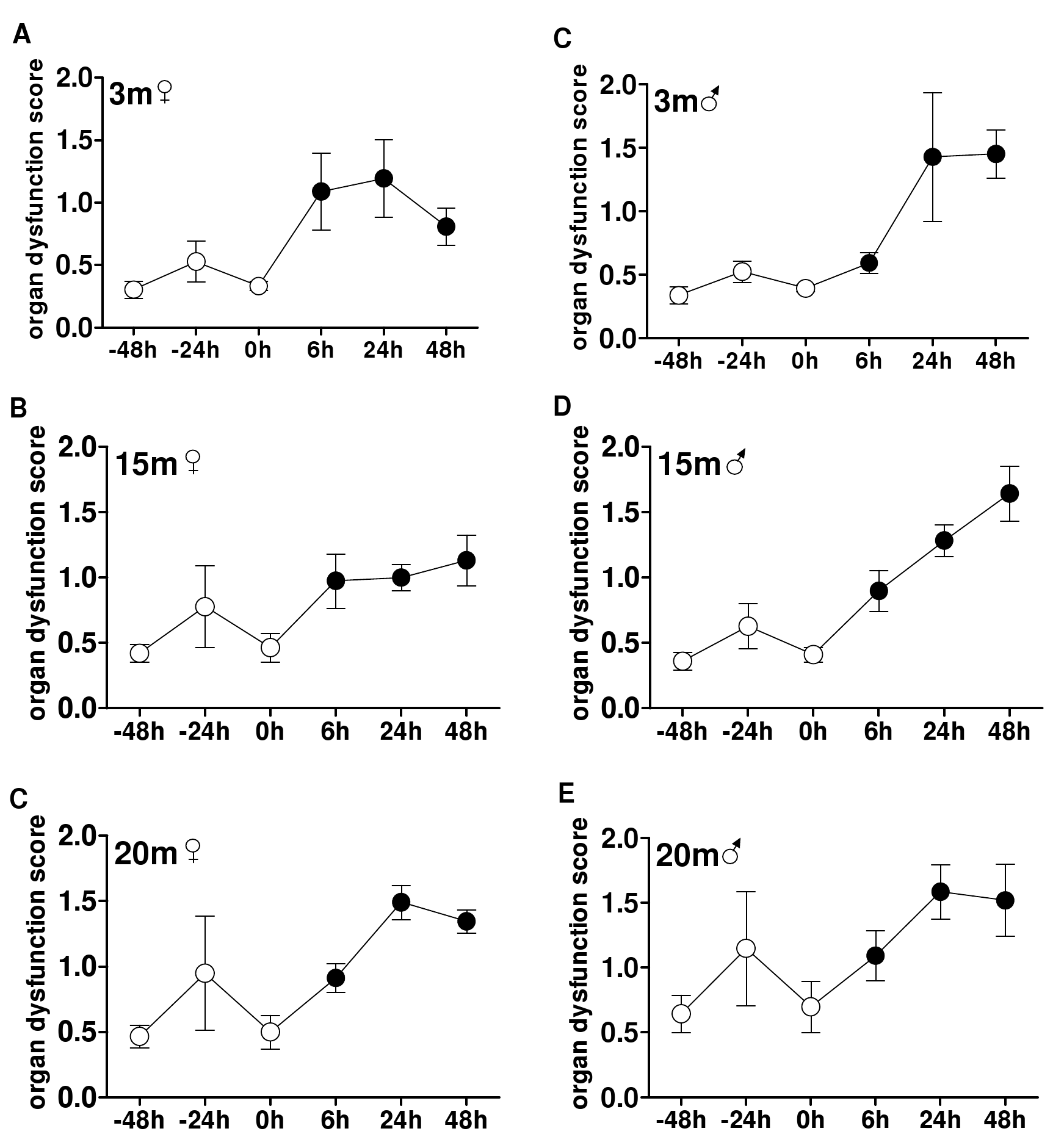

Supplement: Figure S3 — Comparison of the Composite Organ Dysfunction Score in different age/gender groups across two phases of post-traumatic sepsis. Separate score trajectories in 3 (A and C), 15 (B and D) and 20 (C and E) month old female and male mice at −48 h, −24 h, 0 h prior CLP, and 6 h, 24 h and 48 h post-CLP are provided. To enable an overview of organ dysfunction across the entire TH-CLP period, the same median value (i.e. from the 24 h data set) was used for normalization of each cytokine in both pre-and post-CLP phases. Data presented as mean+SEM. The number of animals/group per time-point is identical with n listed in Figs. 3 and 6. No statistical comparisons among age/gender groups are provided here as they are detailed in the respective Figs. 5 and 7. (TIF) [file pone.0051457.s003.tif]

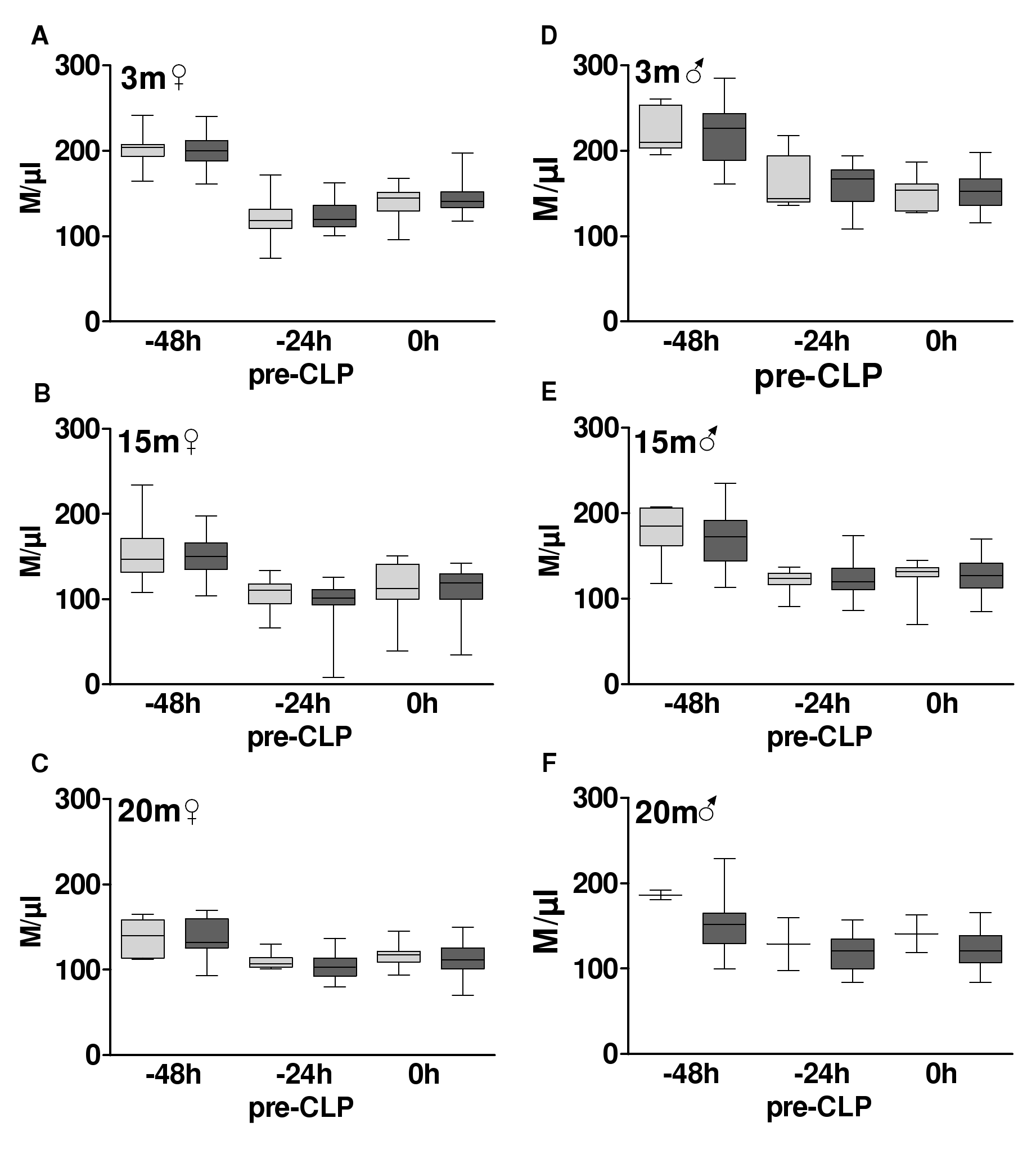

Supplement: Figure S4 — Pre-CLP phase: plasma glucose levels in different age/gender groups. A–F. Glucose plasma levels at −48 h, −24 h and 0 h prior to CLP. SUR = alive on day 16, DIE = died until day 16 post-TH. Data presented as mean+SD. Dotted line represents normal values. In 3 m♀ SUR n≥15, in DIE n≥18 at all time points. In 3 m♂ SUR n = 7, in DIE n = 18 at all time points. In 15 m♀ SUR n≥23, in DIE n = 51 at all time points. In 15 m♂ SUR n≥8, in DIE n≥41 at all time points. In 20 m♀ n = 8, in DIE n = 23 at all time points. In 20 m♂ SUR n = 2, in DIE n = 23 at all time points.*p<0.05 (TIF) [file pone.0051457.s004.tif]

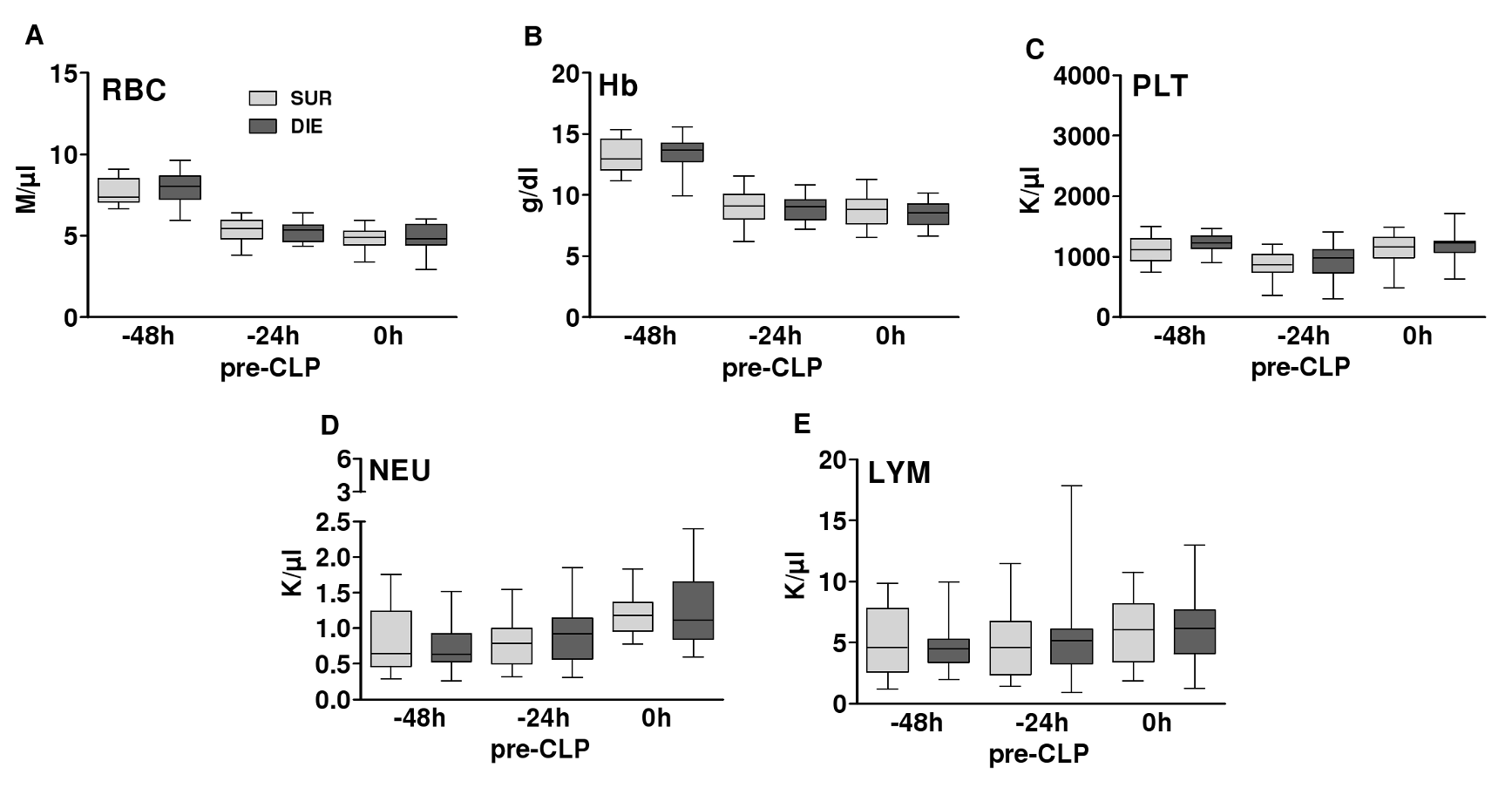

Supplement: Figure S5 — Pre-CLP phase: complete cell count in 3 month old female mice. A–C. Levels of circulating red blood cells (RBC), hemoglobin (Hb) and platelets (PLT) at −48 h, −24 h and 0 h prior CLP. D+E. Circulating neutrophils (NEU) and lymphocytes (LYM) at −48 h, −24 h and 0 h prior to CLP. SUR = alive on day 16, DIE = died until day 16 post-TH. Data presented as box and whiskers (Min, IQR 2, Median, IQR 3, Max). Dotted line represents normal values. In SUR n≥18, in DIE n≥22 at all time points. (TIF) [file pone.0051457.s005.tif]

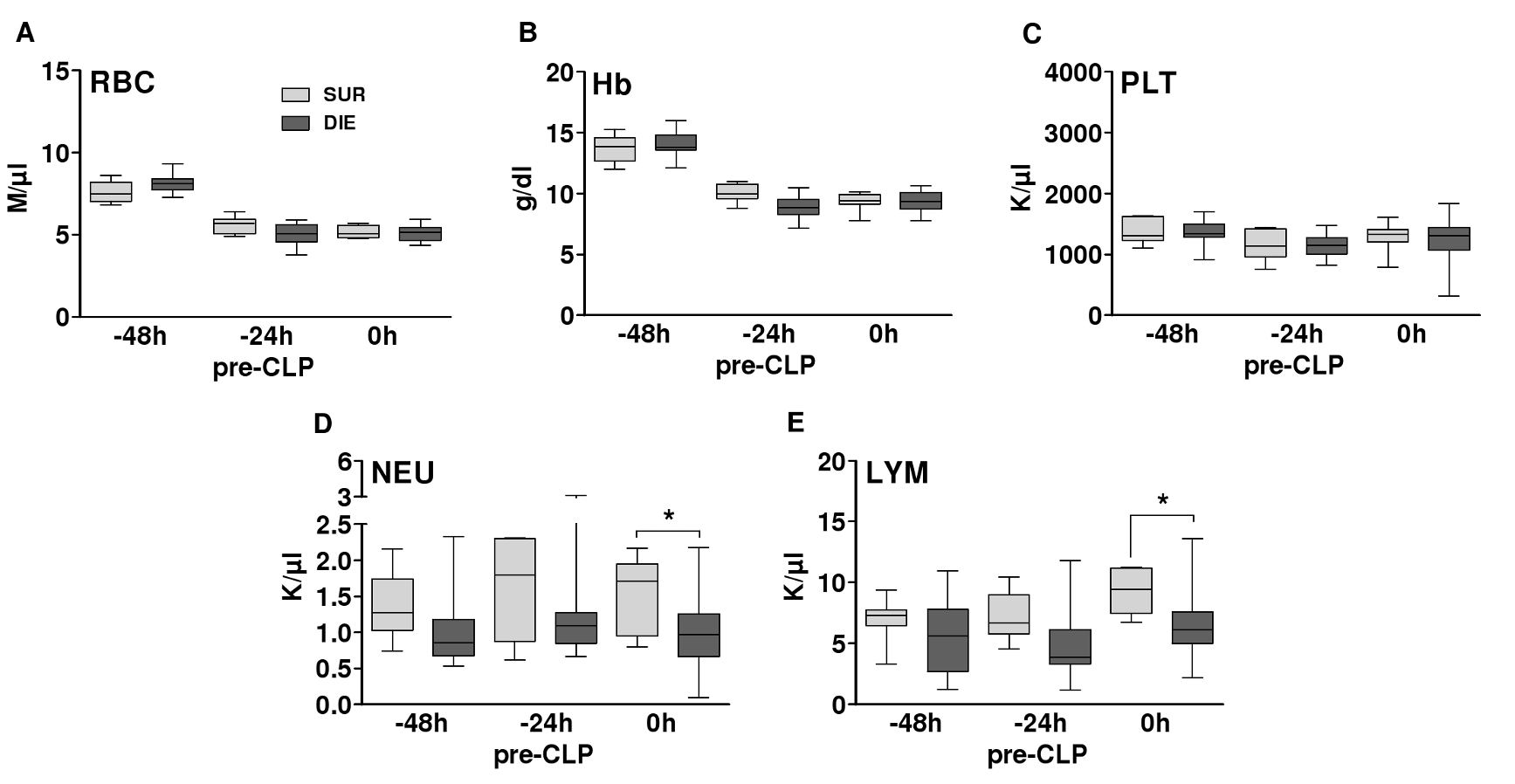

Supplement: Figure S6 — Pre-CLP phase: complete cell count in 3 month old male mice. A–C. Levels of circulating red blood cells (RBC), hemoglobin (Hb) and platelets (PLT) at −48 h, −24 h and 0 h prior CLP. D+E. Circulating neutrophils (NEU) and lymphocytes (LYM) at −48 h, −24 h and 0 h prior to CLP. SUR = alive on day 16, DIE = died until day 16 post-TH. Data presented as box and whiskers (Min, IQR 2, Median, IQR 3, Max). Dotted line represents normal values. In SUR n = 7, in DIE n = 18 at all time points, *p<0.05. (TIF) [file pone.0051457.s006.tif]

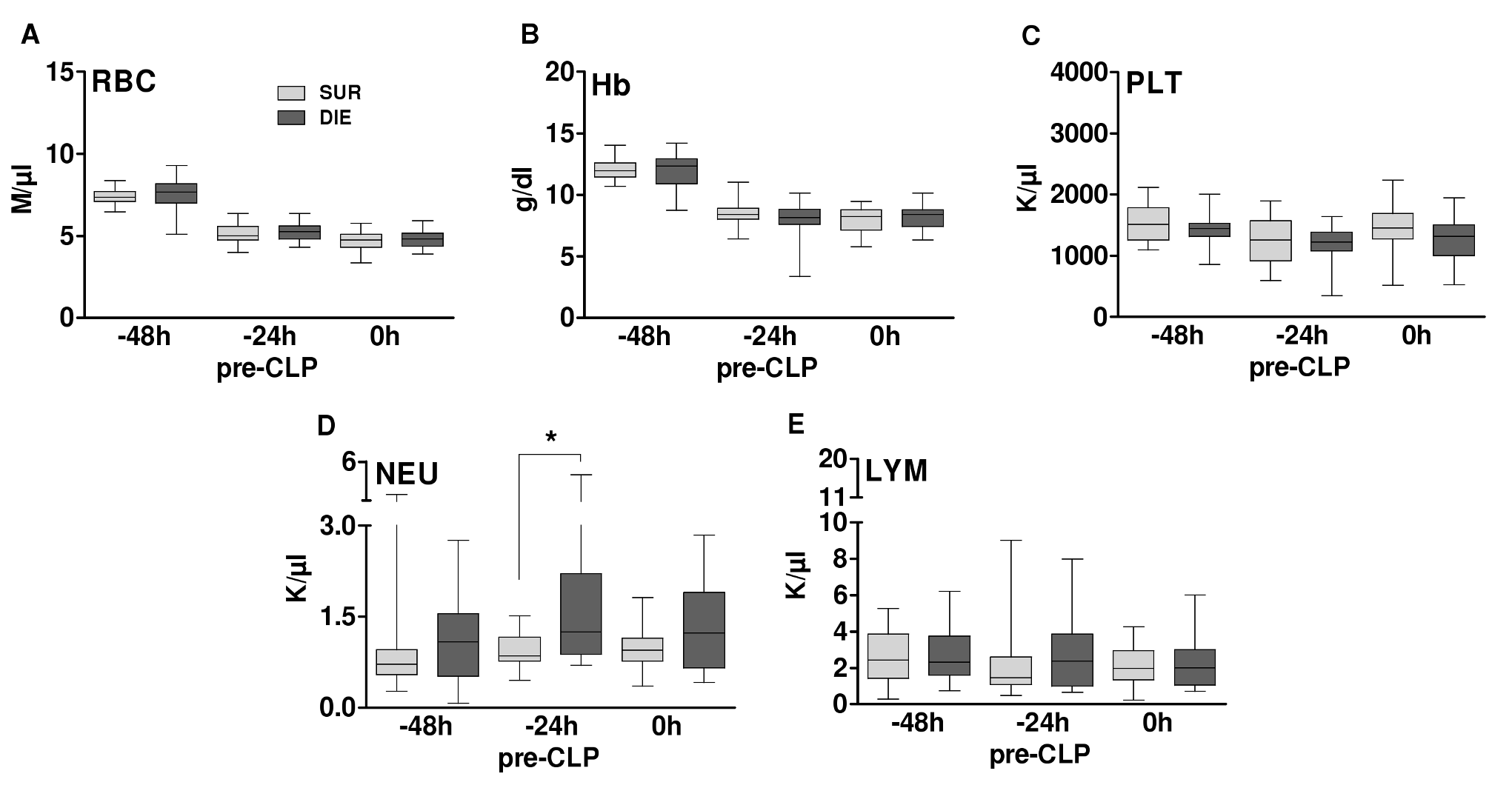

Supplement: Figure S7 — Pre-CLP phase: complete cell count in 15 month old female mice. A–C. Levels of circulating red blood cells (RBC), hemoglobin (Hb) and platelets (PLT) at −48 h, −24 h and 0 h prior CLP. D+E. Circulating neutrophils (NEU) and lymphocytes (LYM) at −48 h, −24 h and 0 h prior to CLP. SUR = alive on day 16, DIE = died until day 16 post-TH. Data presented as box and whiskers (Min, IQR 2, Median, IQR 3, Max). Dotted line represents normal values. In SUR n≥20, in DIE n = 24 at all time points, *p<0.05. (TIF) [file pone.0051457.s007.tif]

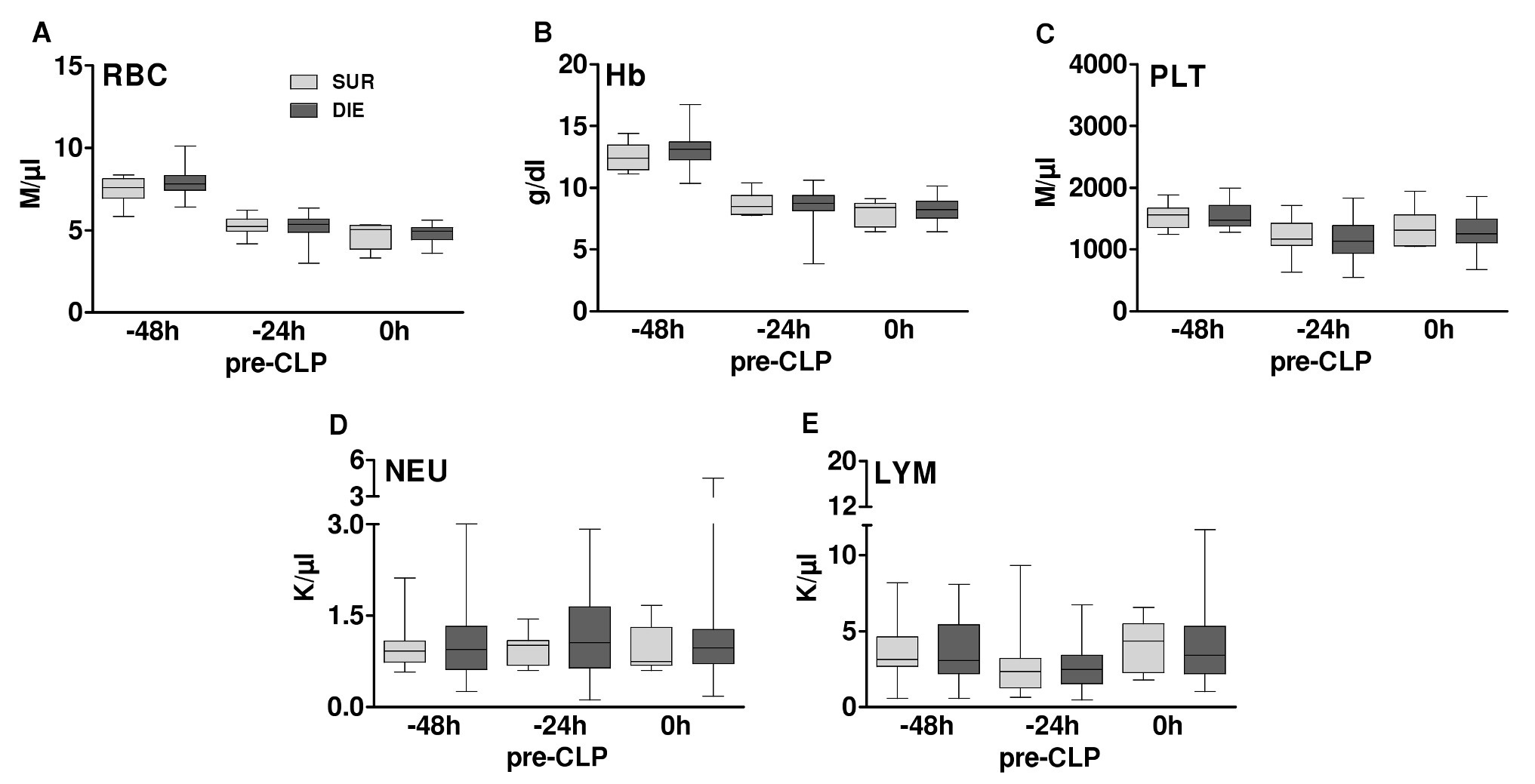

Supplement: Figure S8 — Pre-CLP phase: complete cell count in 15 month old male mice. A–C. Levels of circulating red blood cells (RBC), hemoglobin (Hb) and platelets (PLT) at −48 h, −24 h and 0 h prior CLP. D+E. Circulating neutrophils (NEU) and lymphocytes (LYM) at −48 h, −24 h and 0 h prior to CLP. SUR = alive on day 16, DIE = died until day 16 post-TH. Data presented as box and whiskers (Min, IQR 2, Median, IQR 3, Max). Dotted line represents normal values. In SUR n = 9, in DIE n = at least 37 at all time points. (TIF) [file pone.0051457.s008.tif]

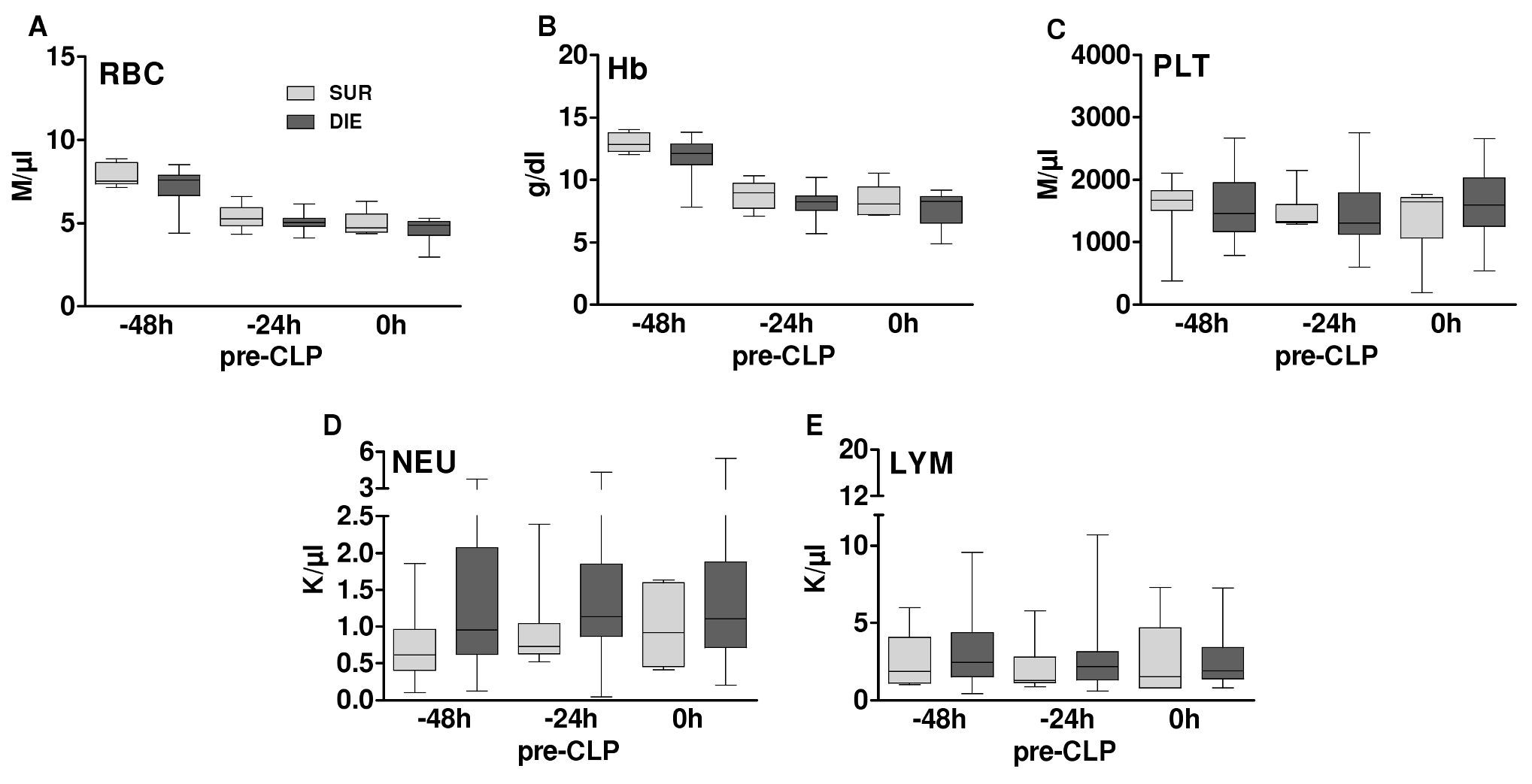

Supplement: Figure S9 — Pre-CLP phase: complete cell count in 20 month old female mice. A–C. Levels of circulating red blood cells (RBC), hemoglobin (Hb) and platelets (PLT) at −48 h, −24 h and 0 h prior CLP. D+E. Circulating neutrophils (NEU) and lymphocytes (LYM) at −48 h, −24 h and 0 h prior to CLP. SUR = alive on day 16, DIE = died until day 16 post-TH. Data presented as box and whiskers (Min, IQR 2, Median, IQR 3, Max). Dotted line represents normal values. In SUR n≥6, in DIE n≥21 at all time points. (TIF) [file pone.0051457.s009.tif]

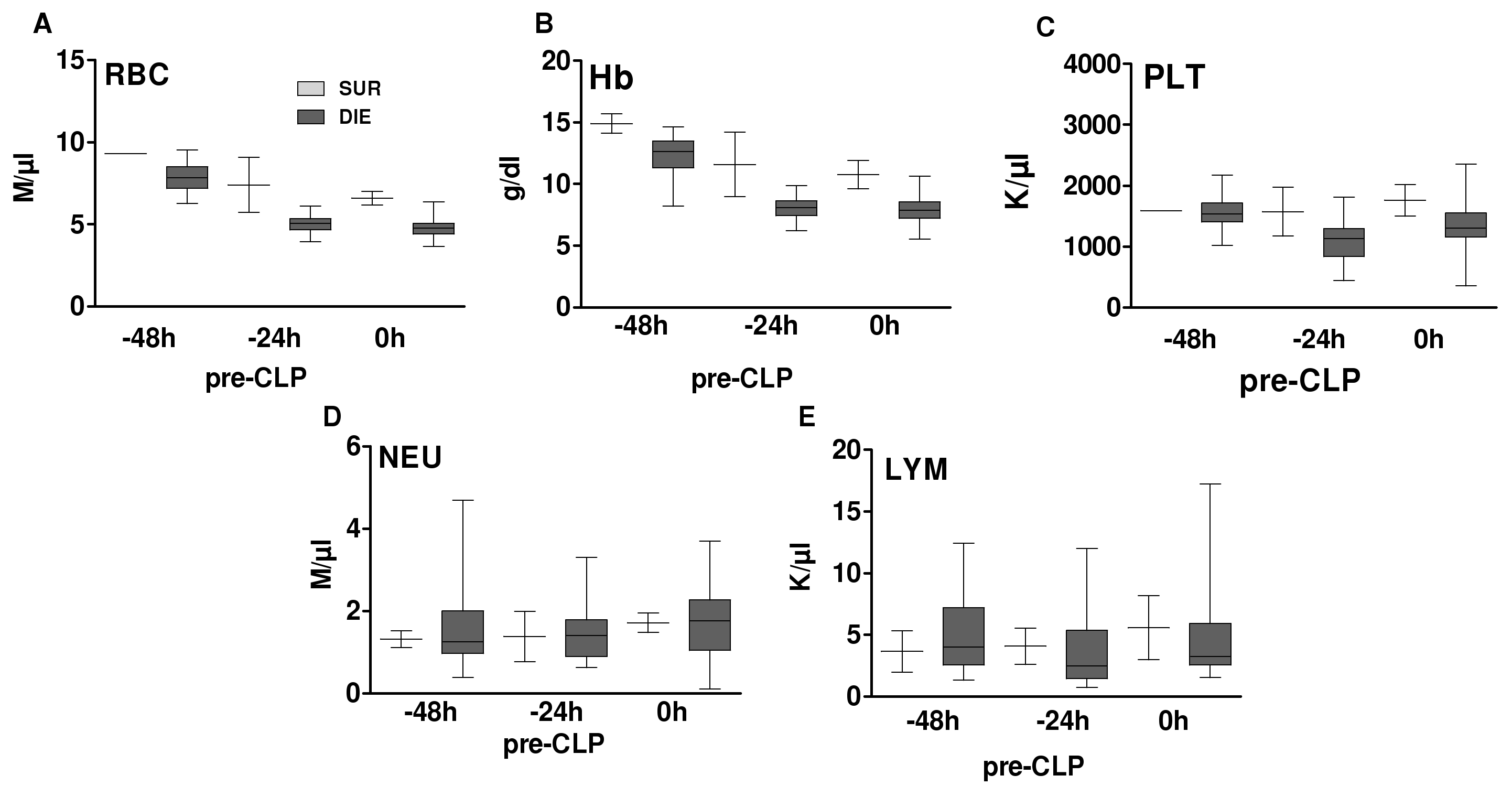

Supplement: Figure S10 — Pre-CLP phase: complete cell count in 20 month old male mice. A–C. Levels of circulating red blood cells (RBC), hemoglobin (Hb) and platelets (PLT) at −48 h, −24 h and 0 h prior CLP. D+E. Circulating neutrophils (NEU) and lymphocytes (LYM) at −48 h, −24 h and 0 h prior to CLP. SUR = alive on day 16, DIE = died until day 16 post-TH. Data presented as box and whiskers (Min, IQR 2, Median, IQR 3, Max). Dotted line represents normal values. In SUR n = 2, in DIE n≥28 at all time points. (TIF) [file pone.0051457.s010.tif]

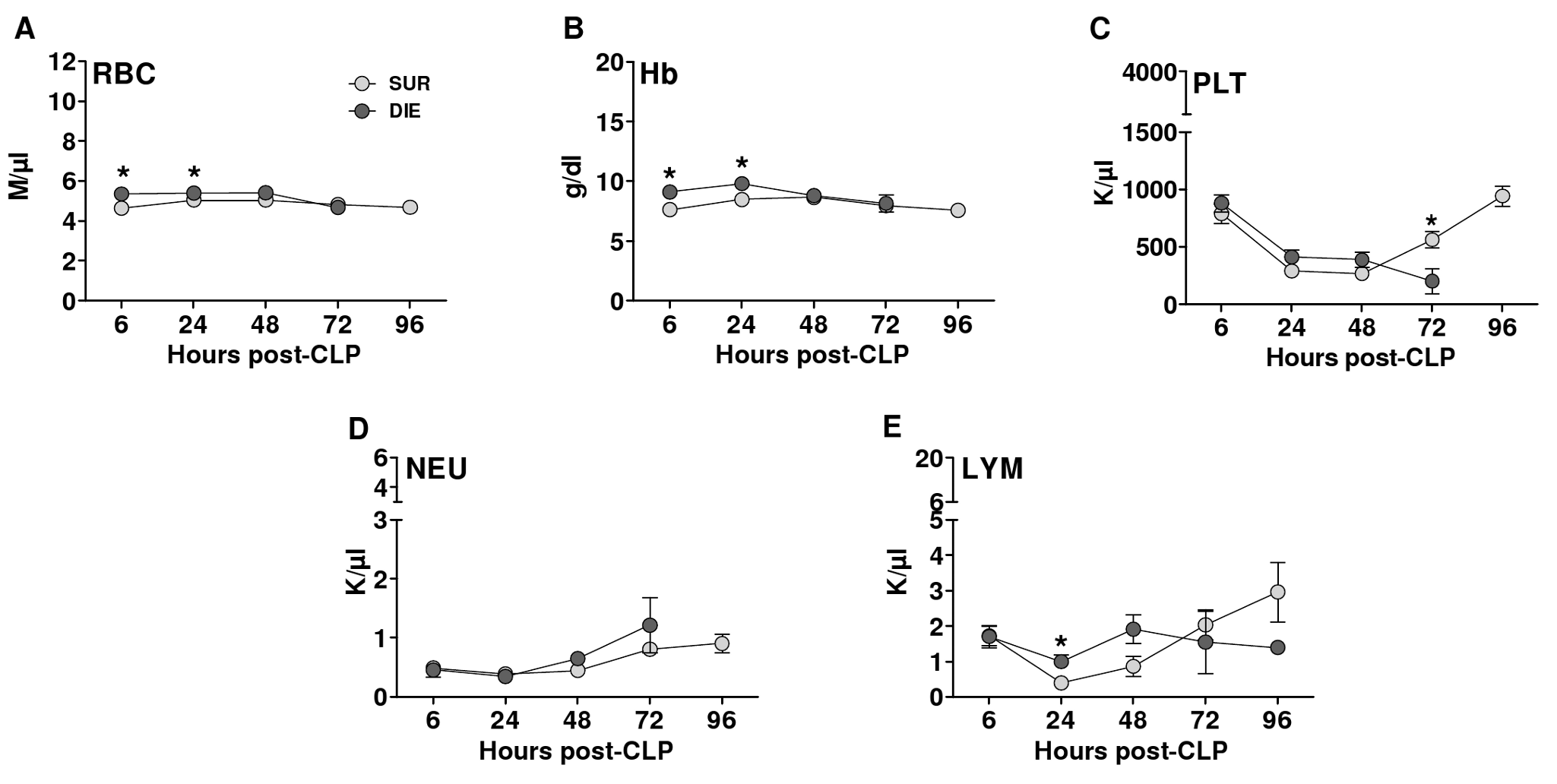

Supplement: Figure S11 — Post-CLP phase: complete cell count in 3 month old female mice. A–C. Levels of circulating red blood cells (RBC), hemoglobin (Hb) and platelets (PLT) at −48 h, −24 h and 0 h prior CLP. D+E. Circulating neutrophils (NEU) and lymphocytes (LYM) at −48 h, −24 h and 0 h prior to CLP. SUR = alive on day 16, DIE = died until day 16 post-TH. Data presented as mean+SEM. Dotted line represents normal values. In SUR n≥12 at all time points, in DIE at 6 h n = 11, at 24 h n = 10, at 48 h n = 5, and at 72 h n = 4. *p<0.05 (TIF) [file pone.0051457.s011.tif]

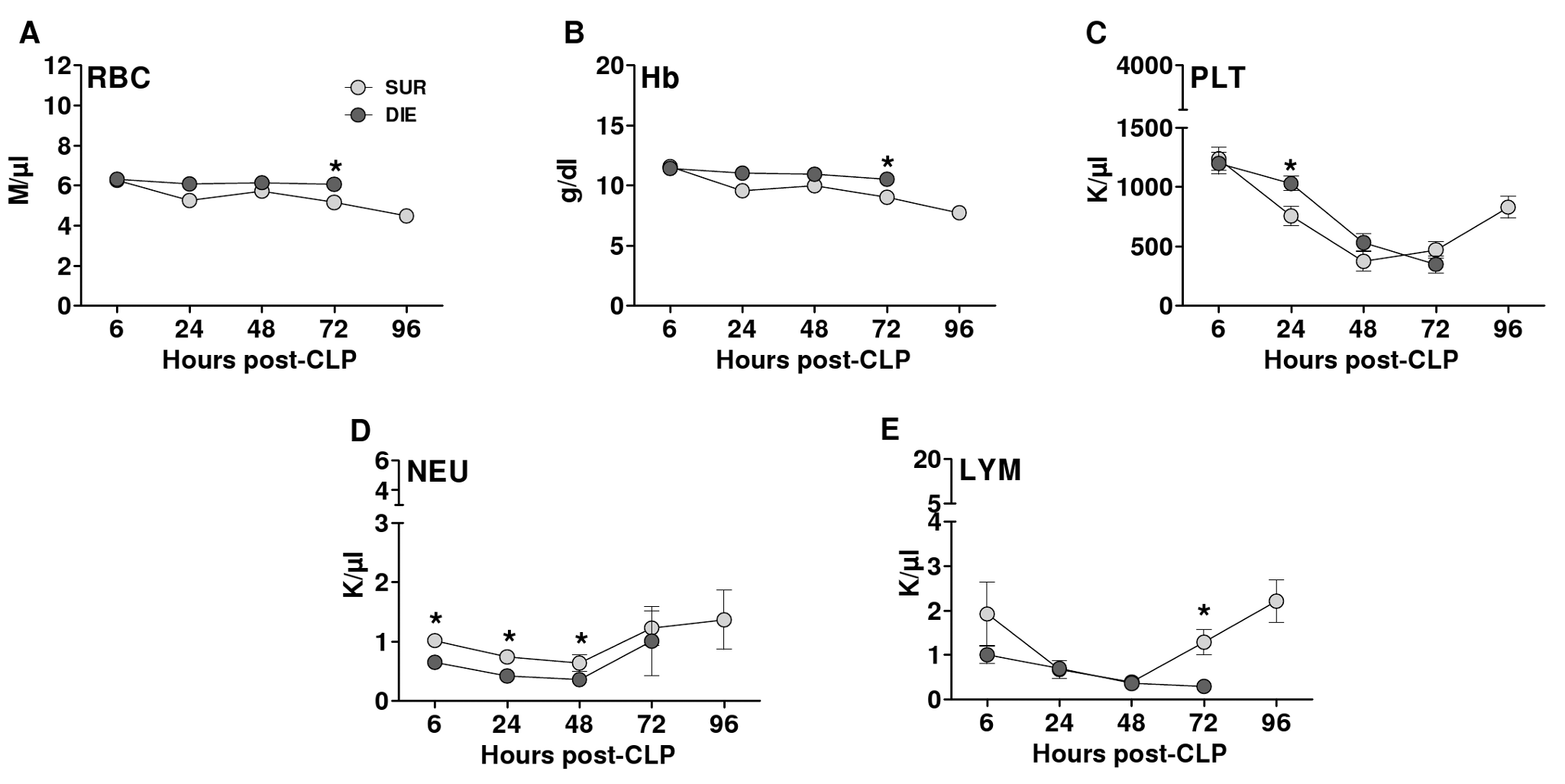

Supplement: Figure S12 — Post-CLP phase: complete cell count in 3 month old male mice. A–C. Levels of circulating red blood cells (RBC), hemoglobin (Hb) and platelets (PLT) at −48 h, −24 h and 0 h prior CLP. D+E. Circulating neutrophils (NEU) and lymphocytes (LYM) at −48 h, −24 h and 0 h prior to CLP. SUR = alive on day 16, DIE = died until day 16 post-TH. Data presented as mean+SEM. Dotted line represents normal values. In SUR n≥7 at all time points, in DIE at 6 h n = 18, at 24 h n = 18, at 48 h n = 14, and at 72 h n = 9. *p<0.05. (TIF) [file pone.0051457.s012.tif]

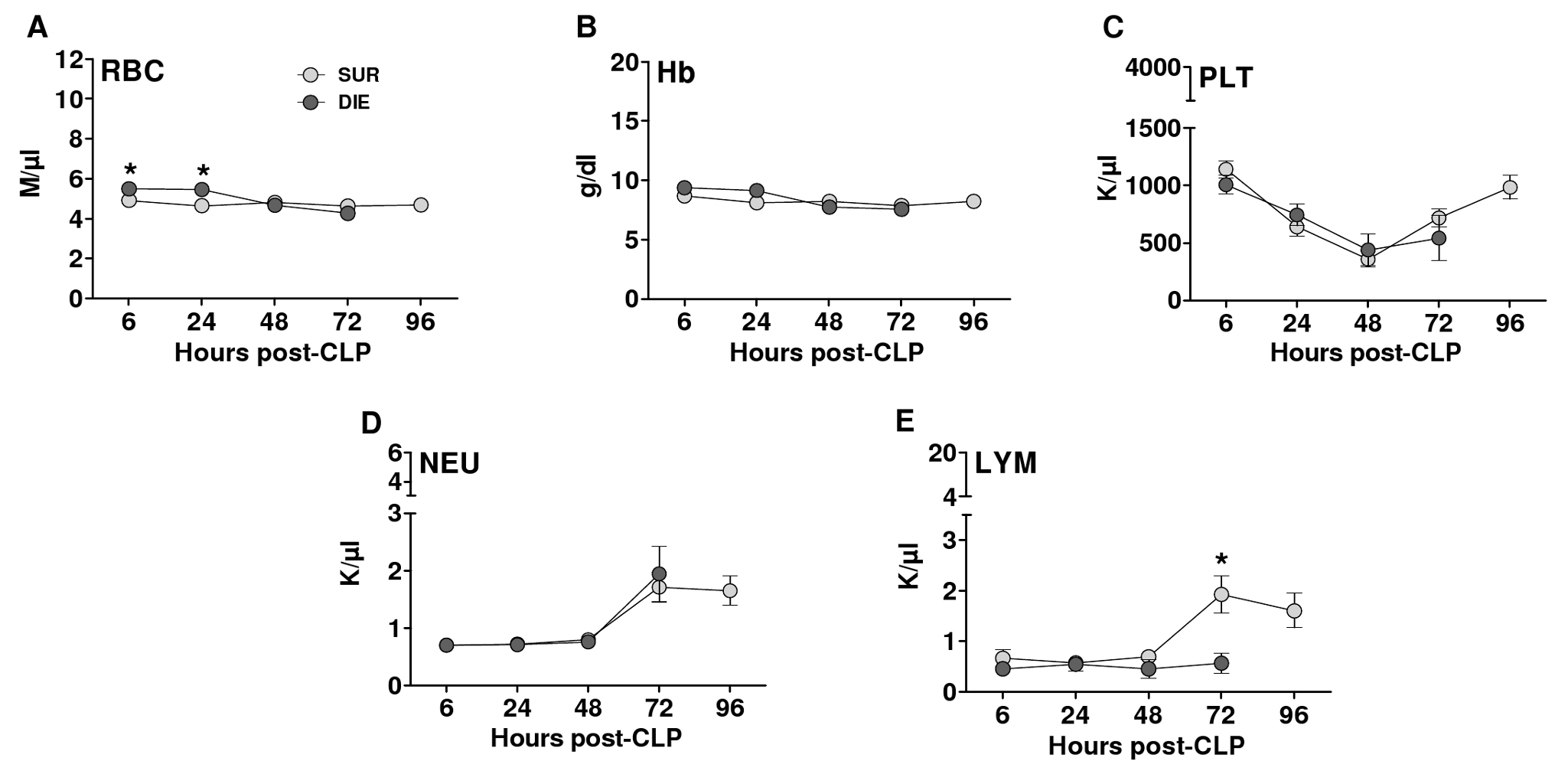

Supplement: Figure S13 — Post-CLP phase: complete cell count in 15 month old female mice. A–C. Levels of circulating red blood cells (RBC), hemoglobin (Hb) and platelets (PLT) at −48 h, −24 h and 0 h prior CLP. D+E. Circulating neutrophils (NEU) and lymphocytes (LYM) at −48 h, −24 h and 0 h prior to CLP. SUR = alive on day 16, DIE = died until day 16 post-TH. Data presented as mean+SEM. Dotted line represents normal values. In SUR n≥17 at all time points, in DIE at 6 h n = 26, at 24 h n = 14, at 48 h n = 10, and at 72 h n = 4. *p<0.05 (TIF) [file pone.0051457.s013.tif]

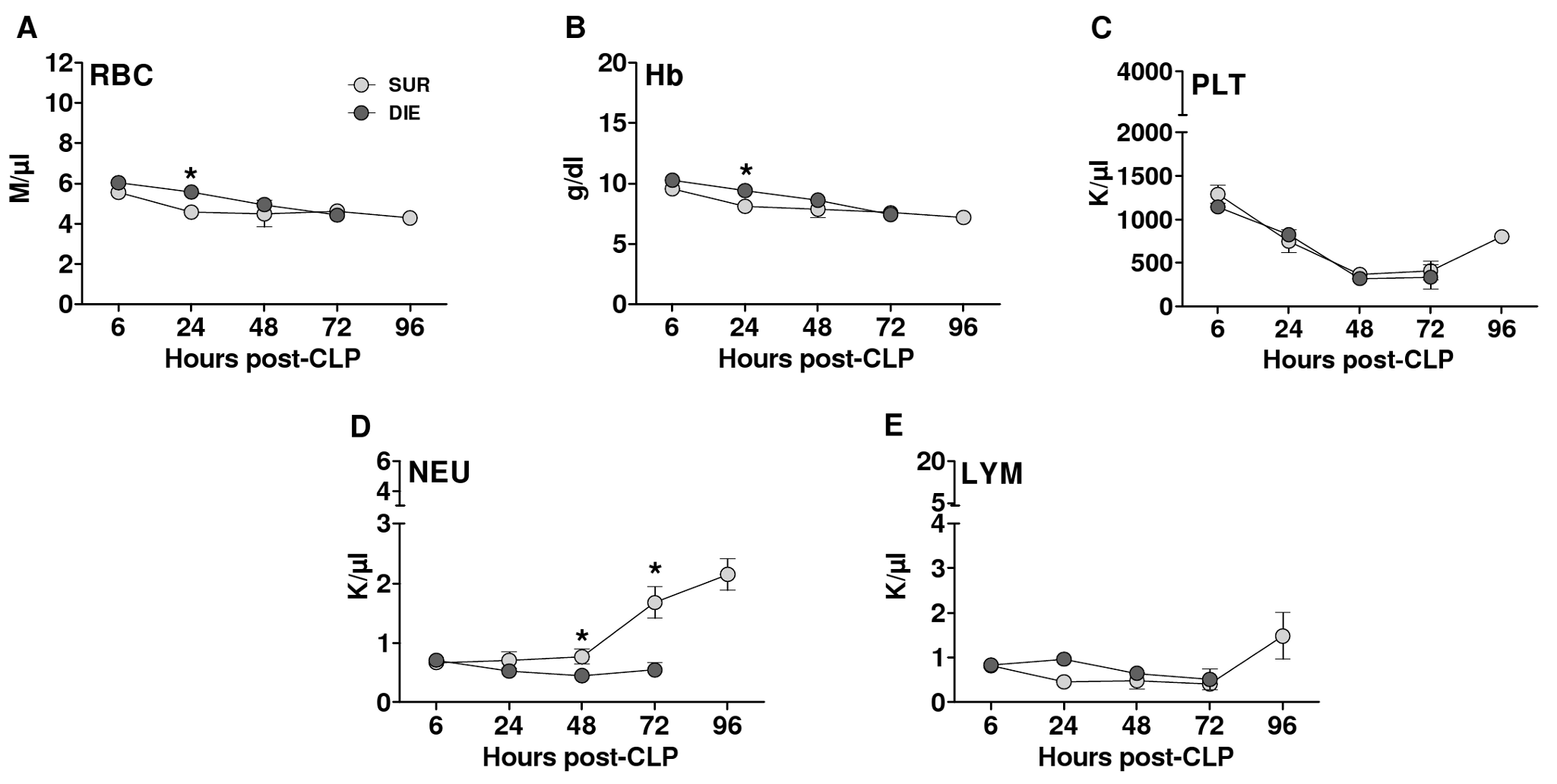

Supplement: Figure S14 — Post-CLP phase: complete cell count in 15 month old male mice. A–C. Levels of circulating red blood cells (RBC), hemoglobin (Hb) and platelets (PLT) at −48 h, −24 h and 0 h prior CLP. D+E. Circulating neutrophils (NEU) and lymphocytes (LYM) at −48 h, −24 h and 0 h prior to CLP. SUR = alive on day 16, DIE = died until day 16 post-TH. Data presented as mean+SEM. Dotted line represents normal values. In SUR n≥8 at all time points, in DIE at 6 h n = 40, at 24 h n = 39, at 48 h n = 22, and at 72 h n = 10. *p<0.05 (TIF) [file pone.0051457.s014.tif]

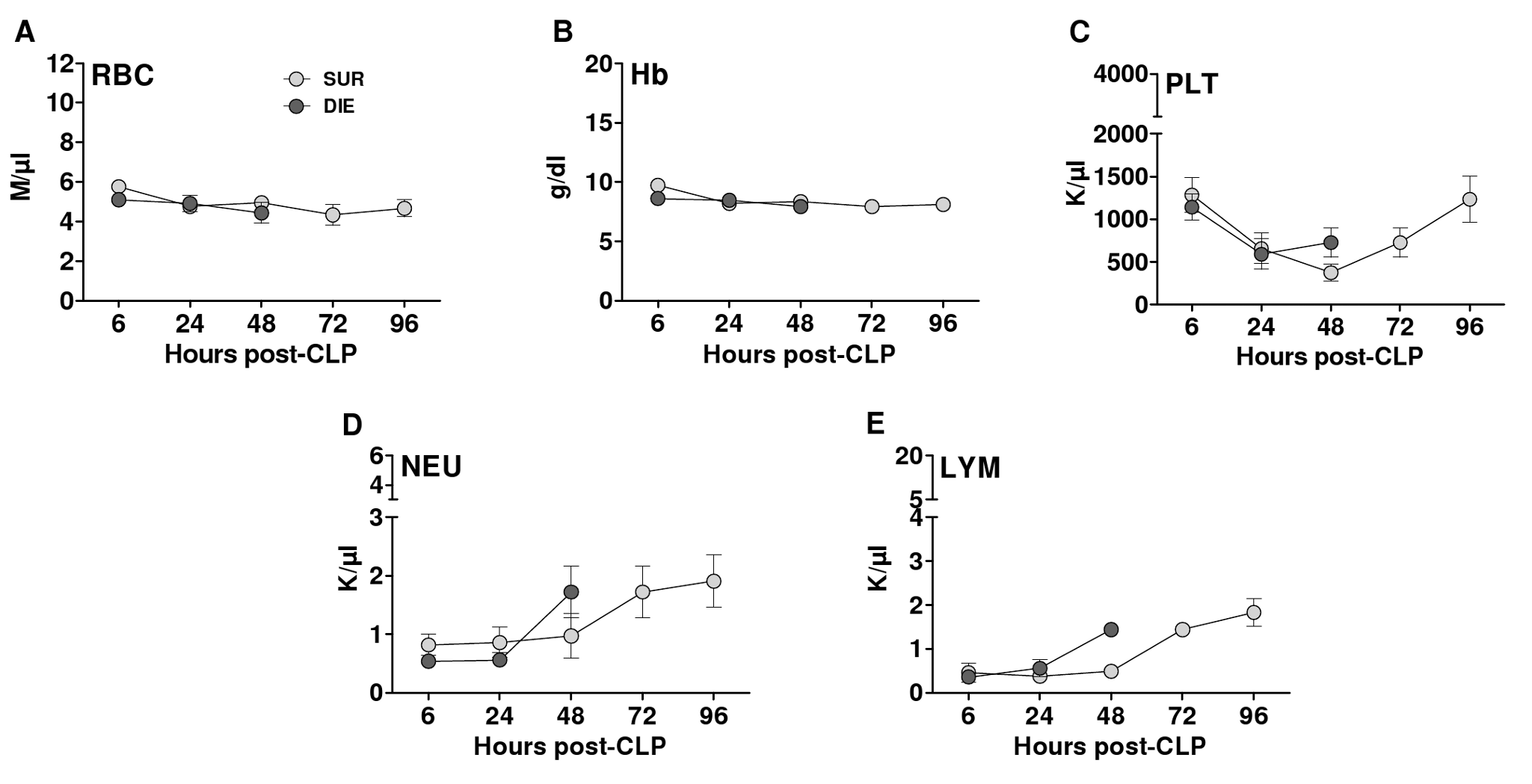

Supplement: Figure S15 — Post-CLP phase: complete cell count in 20 month old female mice. A–C. Levels of circulating red blood cells (RBC), hemoglobin (Hb) and platelets (PLT) at −48 h, −24 h and 0 h prior CLP. D+E. Circulating neutrophils (NEU) and lymphocytes (LYM) at −48 h, −24 h and 0 h prior to CLP. SUR = alive on day 16, DIE = died until day 16 post-TH. Data presented as mean+SEM. Dotted line represents normal values. In SUR n≥8 at all time points, in DIE at 6 h n = 21, at 24 h n = 14, at 48 h n = 6. (TIF) [file pone.0051457.s015.tif]

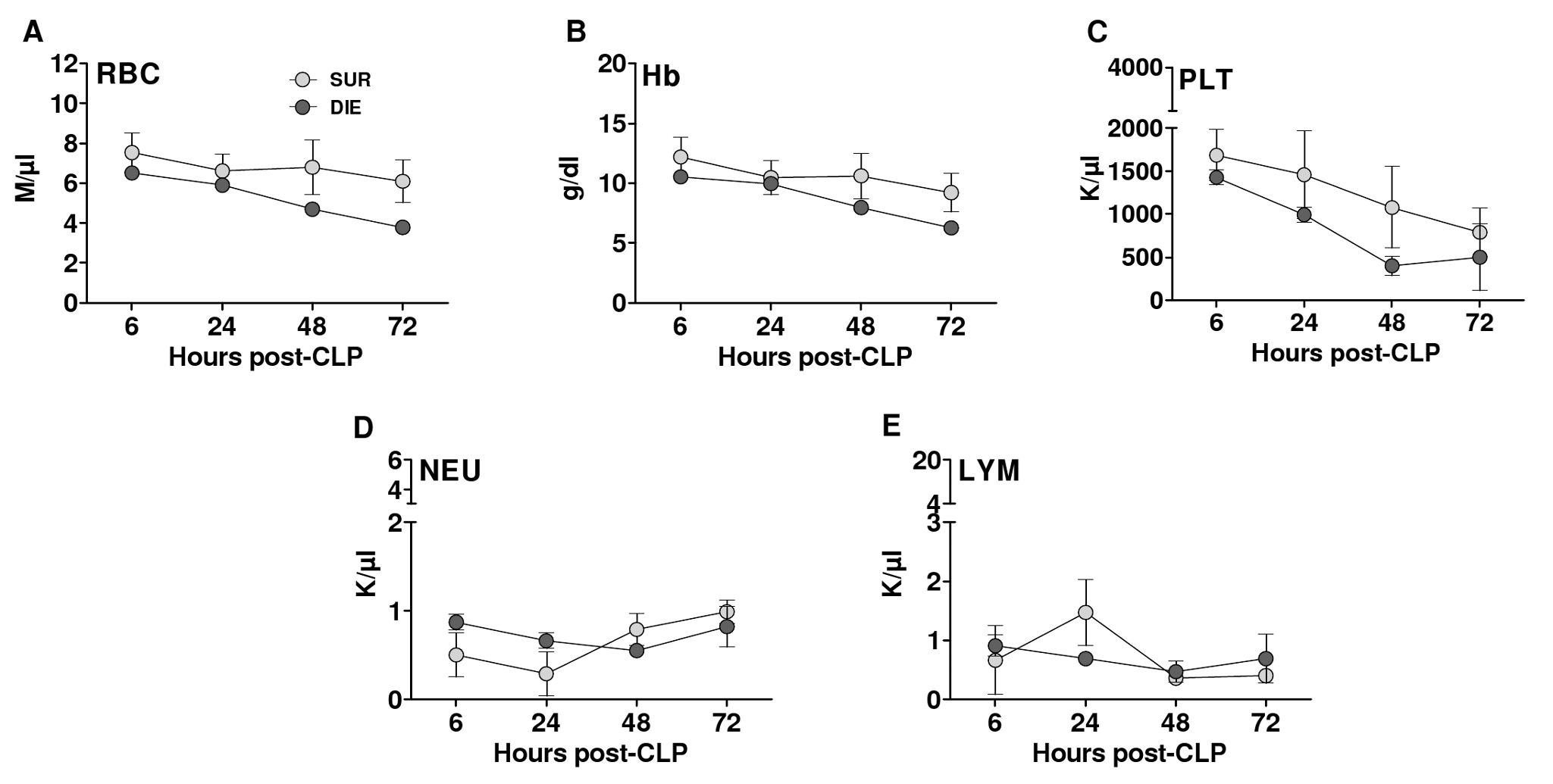

Supplement: Figure S16 — Post-CLP phase: complete cell count in 20 month old male mice. A–C. Levels of circulating red blood cells (RBC), hemoglobin (Hb) and platelets (PLT) at −48 h, −24 h and 0 h prior CLP. D+E. Circulating neutrophils (NEU) and lymphocytes (LYM) at −48 h, −24 h and 0 h prior to CLP. SUR = alive on day 16, DIE = died until day 16 post-TH. Data presented as mean+SEM. Dotted line represents normal values. In SUR n = 2 at all time points, in DIE at 6 h n = 25, at 24 h n = 20, at 48 h n = 10, and at 72 h n = 2 (TIF) [file pone.0051457.s016.tif]
